# Automated and High-Throughput Phase Separation Control for Supramolecular Polymer Blends Enabled by Machine Learning

**DOI:** 10.1021/jacsau.6c00041

**Published:** 2026-05-29

**Authors:** Yunfei Wang, Daniel Struble, Saroj Upreti, Zongliang Xie, Ka Hung Chan, Yi Liu, Chenhui Zhu, Paul Ashby, Wenjie Xia, Derek Patton, Boran Ma, Xiaodan Gu

**Affiliations:** † School of Polymer Science and Engineering, Center for Optoelectronic Materials and Devices, 5104 the University of Southern Mississippi, Hattiesburg, Mississippi 39406, United States; ‡ Advanced Light Source, 1666 Lawrence Berkeley National Laboratory, Berkeley, California 94720, United States; § The Molecular Foundry, Lawrence Berkeley National Laboratory, Berkeley, California 94720, United States; ∥ Materials Sciences Division, Lawrence Berkeley National Laboratory, Berkeley, California 94720, United States; ⊥ Department of Aerospace Engineering, 1177 Iowa State University, Ames, Iowa 50011, United States

**Keywords:** automation and high-throughput materials discovery, high-throughput characterization, ML-guided polymer design, supramolecular polymer blends

## Abstract

Supramolecular polymer blends (SPBs) offer tunable morphologies that dictate their macroscopic properties, yet their rational design is limited by the absence of predictive structure–morphology models. Here, we introduce a data-driven high-throughput workflow that integrates modular polymer synthesis, robotic formulation, automated morphology characterization, and machine learning (ML) for accelerated SPB discovery. Using a plug-and-play synthetic strategy, 33 hydrogen-bonding end-functional homopolymers were prepared and orthogonally combined to generate 260 SPBs in 1 day. A fully automated atomic force microscopy (AFM) pipeline enabled systematic imaging, producing 2340 morphology data sets with minimal human intervention. Domain spacings were extracted through complementary image-processing methods and used to train ML models. A support vector regression (SVR) model accurately predicted target phase-separation sizes (50, 100, and 150 nm), which were experimentally validated. This work demonstrates the power of coupling high-throughput experimentation with ML to accelerate morphology discovery and provides one of the first large-scale experimental data sets for supramolecular polymer systems.

## Graphical abstract

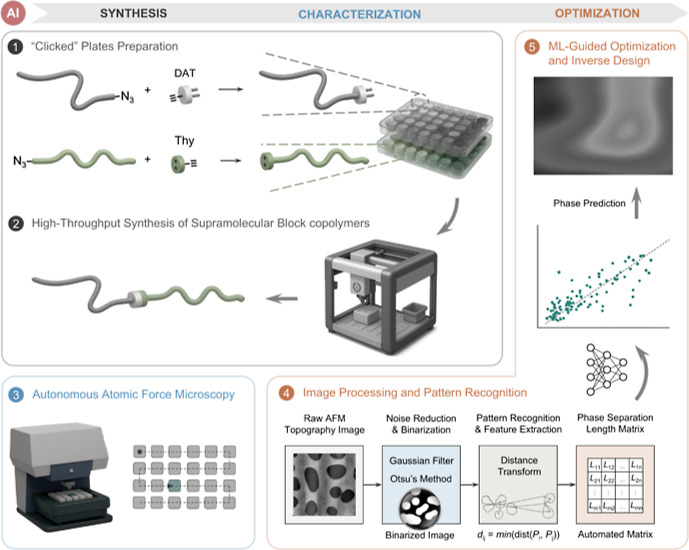

## Introduction

Supramolecular chemistry provides a transformative route to functional materials by using dynamic, noncovalent interactions to direct the bottom-up assembly of molecules.^1–9^ This approach yields materials with programmable, biomimetic properties, such as adaptivity and self-healing, offering capabilities beyond the reach of traditional covalent polymers. A leading platform in this domain is supramolecular polymer blends (SPBs), which self-assemble from polymer chains or small-molecule building blocks linked by specific interactions, such as hydrogen bonds, host–guest recognition, and metal–ligand coordination.^10–16^ The resulting morphology is of paramount importance, as it directly governs the macroscopic optical, mechanical, and electronic properties of the material. Despite this immense potential, a fundamental bottleneck hinders progress: predicting the final morphology from the constituent molecular components remains a formidable scientific challenge. This knowledge gap makes the systematic exploration of the vast design space an inherently low-throughput process, impeding the rational design of next-generation functional materials enabled by supramolecular chemistry.

Artificial Intelligence and Machine Learning (AI/ML) technologies present a promising solution for accelerating polymer research by identifying chemical structure–morphology relationships.^17–22^ For instance, previous studies using ML have successfully predicted morphological outcomes in grafted block copolymer composites.^23^ However, robust predictive models require extensive experimental data sets. Alternatively, many studies rely on historical data sets accumulated over several years or simulated data, both of which have inherent limitations in accuracy, reproducibility, and applicability to real-world scenarios. Therefore, developing automated, high-throughput synthetic methods for generating comprehensive SPBs remains an urgent need.

Traditionally, studies on self-assembly of SPBs have been largely limited in scope, with only a few compositions investigated in each work. In most cases, SPBs were synthesized individually, which is highly inefficient and insufficient for establishing comprehensive structure–property relationships. For example, Feldman et al. examined the morphology and stability of poly­(*n*-butyl acrylate)-poly­(benzyl methacrylate) (PnBA-PbnMA) SPBs, but only a single molecular weight (*MW*) pair of PnBA and PbnMA was reported.^24^ Similarly, Bhaumik et al. studied the nanostructure of polystyrene–polyisoprene (PS–PI) SPBs, yet only three *MW* pairs were explored.^16^ Xie et al. investigated the effect of *M*W on the morphology and self-assembly behavior of PS-polydimethylsiloxane (PS–PDMS) SPBs connected by ionic interactions; however, the data set was limited to one *MW* of PDMS combined with two *MW*s of PS, with additional trends inferred from simulations, which may introduce inaccuracies.^25^

In this study, we developed a two-step, plug-and-play modular synthesis and fabrication strategy to enable rapid, high-throughput preparation of SPBs for an ML-driven structure–property relationship study (Scheme 1). A modular approach was first developed to synthesize hydrogen-bonding end-functional homopolymer precursors and subsequently orthogonally pair them to assemble SPBs. From only 33 homopolymer precursors, a library of 260 SPBs was fabricated within a single day using automated blending. Morphological characterization was then carried out using our newly developed automated atomic force microscopy (AFM) protocol, yielding thousands of nanoscopic images of samples within 7 days with little human intervention. The resulting images were systematically processed with custom Python scripts, and the extracted domain spacings were successfully applied to morphology prediction and inverse design through ML. The entire workflow required only several months from synthesis to predictive modeling, demonstrating a significant improvement in the efficiency of material design. Using this new platform, we were able to predict, control, and generate the phase separation size of the binary SPB, where in this case, 50, 100, and 150 nm phase separation sizes were demonstrated. The platform thus presents a remarkable advancement in automated material synthesis, characterization, and predictive capability, demonstrating the potential of AI/ML-driven methodologies to accelerate polymer research and discovery. To our knowledge, this represents the first generation of hundreds of reliable, experimentally derived data sets specifically designed to support ML in supramolecular polymer research. The reported publicly accessible database, together with an automated material discovery workflow, significantly advances the future of data-driven polymer design.

**Scheme 1:**
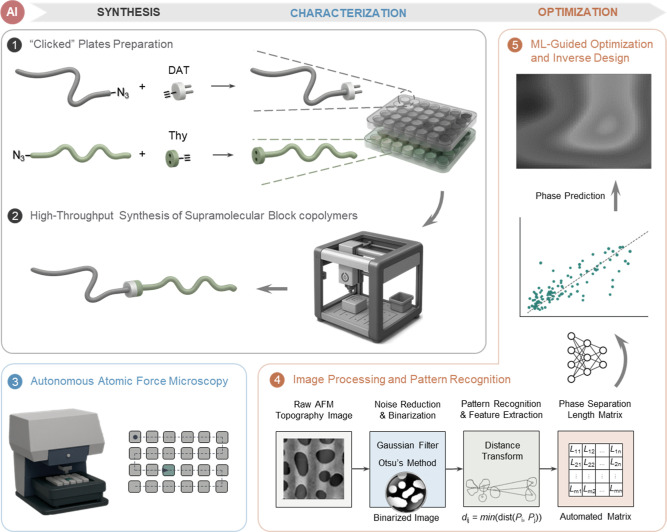
Overview of Automated, High-Throughput Phase Separation Control in Supramolecular Polymer Blends Enabled by Machine Learning^a^

a The workflow integrates (1) click chemistry for synthesizing homopolymers with hydrogen-bonding chain ends, (2) high-throughput combinatorial formulation, (3) AFM characterization of supramolecular polymer blends, (4) large-scale data reduction and analysis, and (5) machine learning to establish structure–processing–morphology relationships.

## Results and Discussion

The two-step, plug-and-play modular synthesis strategy developed for the high-throughput fabrication of SPBs includes (1) the modular synthesis of homopolymer precursors bearing complementary hydrogen-bonding end-groups and (2) the modular assembly of SPBs by pairing any two precursors with orthogonal binding motifs. The hydrogen-bonding pair 2,6-diaminotriazine (DAT) and thymine (Thy) was selected as the terminal functional group due to their strong and selective intermolecular interactions.^11,12^

### Modular Synthesis of Comprehensive Hydrogen-Bonding End-Functional Homopolymer Precursors with Various Structures and MWs

A ‘Lego-like’ modular synthesis approach was adopted to construct a broad library of DAT- and Thy-terminated homopolymer precursors, achieved by integrating hydrogen-bonding motifs into polymer modules via highly efficient Copper-Catalyzed Azide–Alkyne Cycloaddition (CuAAC) click chemistry (Figure 1a). Specifically, alkyne-functionalized DAT and Thy were initially synthesized as hydrogen-bonding modules. Concurrently, azide-functionalized homopolymers and chain transfer agents (CTAs) were prepared as complementary building blocks. These components were subsequently coupled through the CuAAC “click” reaction to generate either DAT/Thy-functionalized homopolymers or functionalized CTAs. The CTAs were further used to produce homopolymers via reversible addition–fragmentation chain transfer (RAFT) polymerization. With these synthetic strategies, a diverse library of homopolymer precursors with varied architecture and *MW*s was obtained, including poly­(oligo­(ethylene glycol) methacrylate)-DAT (POEGMA-DAT), polyethylene glycol-DAT (PEG-DAT), poly­(methyl methacrylate)-DAT (PMMA-DAT), PS-Thy, and PMMA-Thy. All chain-end hydrogen-bonded homopolymers exhibited narrow dispersity (Figure 1b,c, Tables S1–S3). Detailed synthesis protocols, intermediate characterization by ^1^H NMR spectroscopy, and complete molecular weight analyses are provided in the Supporting Information (Figure S1).

**Figure 1:**
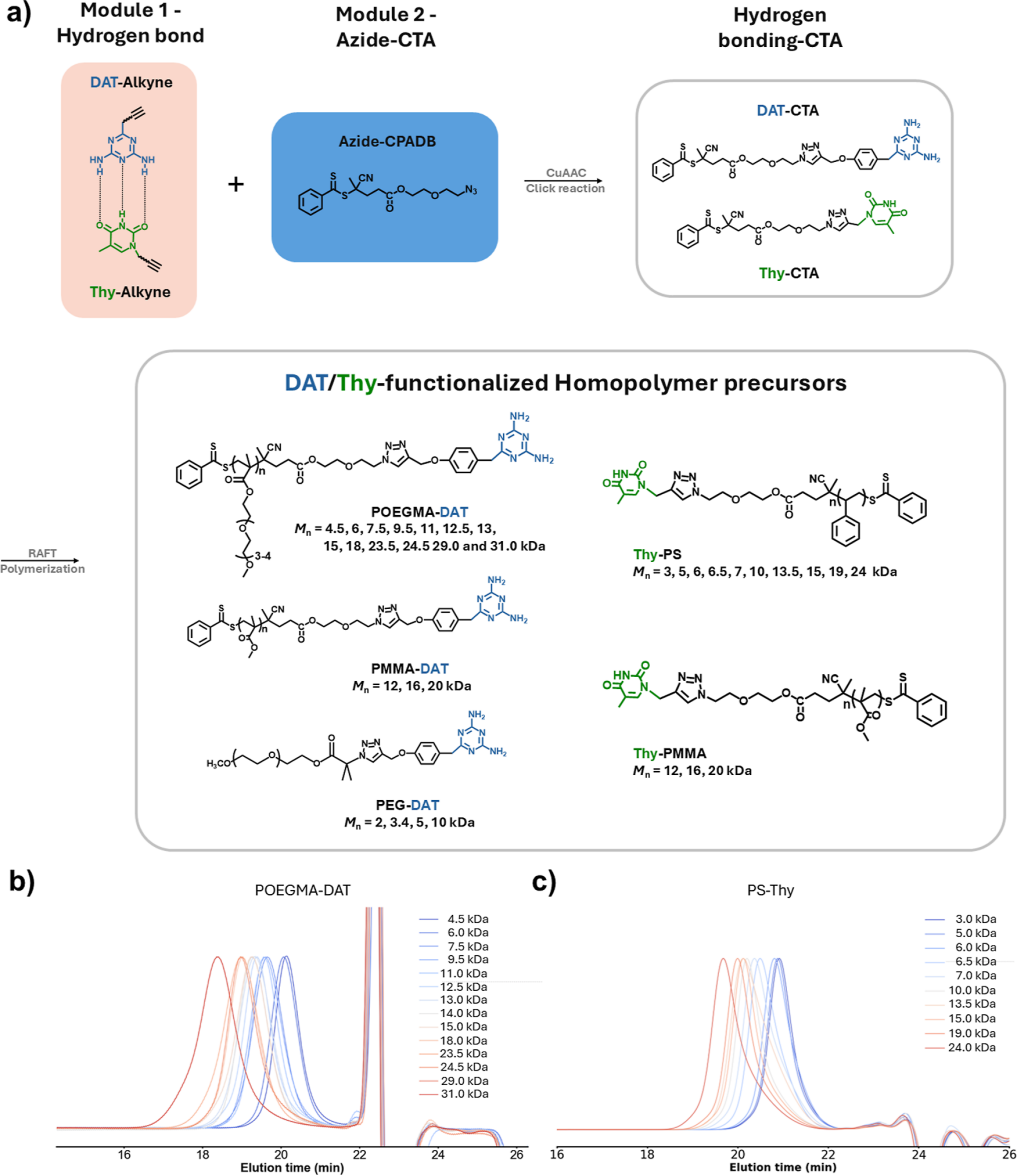
Modular synthesis strategy to construct a comprehensive library of DAT- and Thy-end-functionalized homopolymer precursors, including DAT-functionalized POEGMA, PMMA, and PEG, along with Thy-functionalized PS and PMMA. (a) To prepare these polymers, alkyne-functionalized DAT and Thy derivatives were first synthesized, then coupled with azide-functionalized CTAs through high-efficiency CuAAC click chemistry, producing DAT/Thy-functionalized CTAs. The resulting CTAs were further used in RAFT polymerization to create an expanded library of DAT- and Thy-functional homopolymer precursors. The modular “grafting-to” synthesis method was also applied to build up the DAT/Thy-functional homopolymers, which are shown in Supporting Information. All molecular weights were characterized using GPC. GPC elution curve of (b) POEGMA-DAT in DMF and (c) PS-Thy in THF.

Subsequently, a total of 260 SPBs were prepared in a high-throughput manner using combinatorial chemistry, achieved by the solution formulation of the 33 synthesized homopolymer precursors with a liquid-handling robot. Each DAT-functionalized homopolymer solution was paired with an orthogonal Thy-functionalized homopolymer solution in toluene without additional processing. In contrast to conventional synthesis approaches, the integration of modular synthesis with automated blending dramatically accelerates SPB fabrication, reducing the development timeline from years to months while substantially minimizing manual effort (Figure 2a).

**Figure 2:**
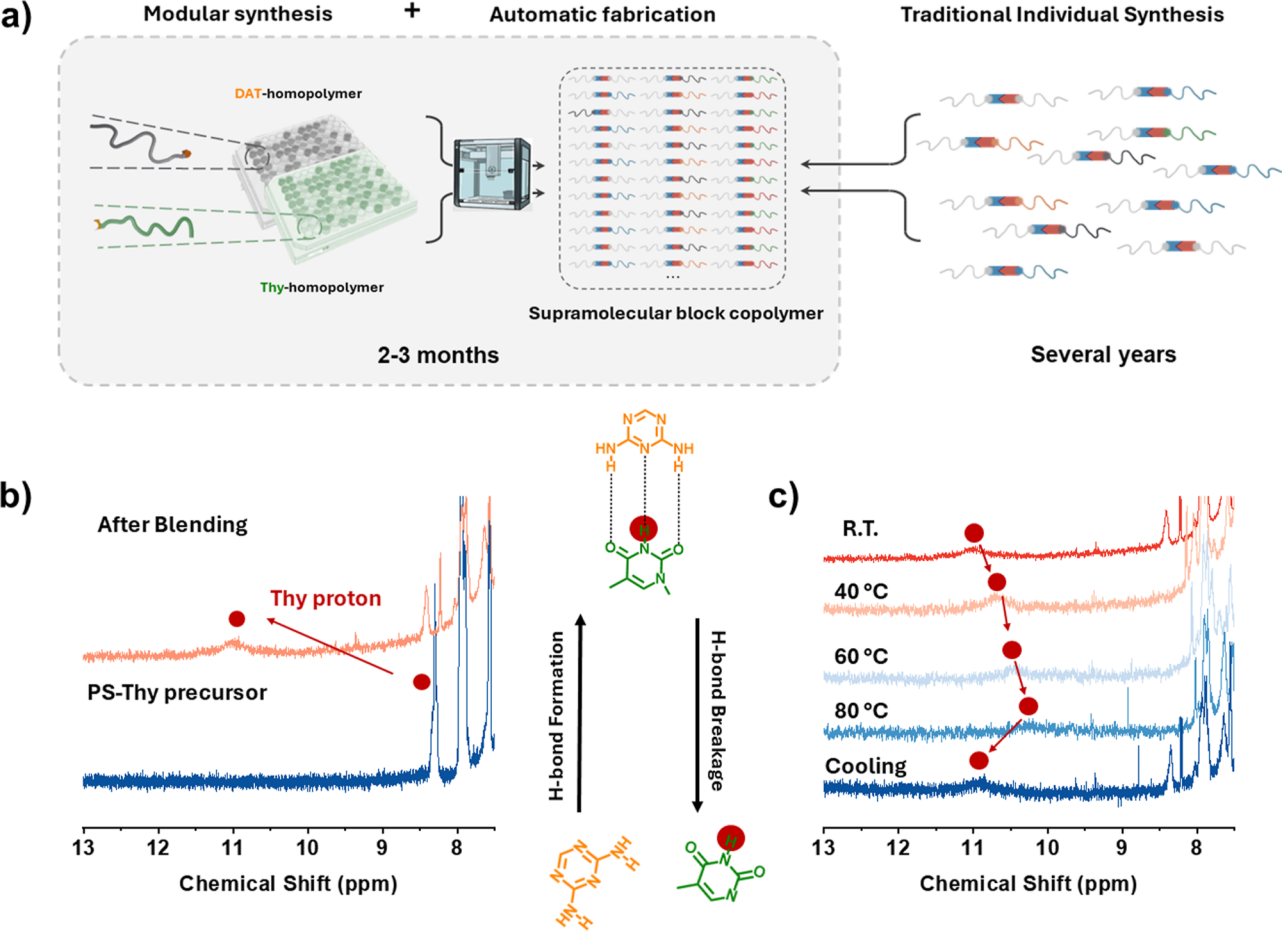
High-throughput combinatorial synthesis of SPB. (a) Automated, high-throughput fabrication of SPB compared with conventional stepwise synthesis. (b) ^1^H NMR spectra of PS-Thy and the POEGMA-DAT/PS-Thy mixture in toluene-*d*_6_. The downfield shifts of the Thy protons upon blending confirm the formation of intermolecular hydrogen-bonding interactions. (c) In situ variable-temperature (VT) ^1^H NMR spectra of POEGMA/PS SPB, recorded during heating from room temperature to 80 °C and subsequent cooling back to room temperature.

The successful formation of binary hydrogen bonding blends was confirmed by ^1^H NMR spectroscopy. Figure 2b shows representative ^1^H NMR spectra of the homopolymer precursor, PS-Thy, and the blended POEGMA-DAT/PS-Thy solution in toluene-*d*_6_. Upon blending, the Thy proton resonance shifted from 8.3 to 11 ppm, indicating the formation of intermolecular hydrogen-bonding interactions, consistent with observation from small-molecule DAT/Thy systems (Figure S2). When the solution was subsequently heated to 40, 60, and 80 °C, the Thy imide proton peak progressively shifted upfield, reflecting the weakening of hydrogen bonding (Figure 2c). Upon cooling to room temperature, the peak shifted back downfield toward its original position, demonstrating reformation of the hydrogen bond. These observations confirm the reversible hydrogen bonding interactions within SPB.

After SPB formulation, all of the samples were spin-coated onto silicon substrates to form polymeric thin films. For each sample, a duplicate was made and immersed in ethanol to remove the POEGMA domain to enhance the height contrast between the POEGMA and PS domains.

### Automated Morphology Characterization and Analysis

To enable high-throughput morphology analysis, we established an automated AFM workflow capable of automated sample alignment, optical image acquisition, tip tuning, and scanning for each sample (Figures 3a and S2). The protocol was implemented on an Asylum Jupiter discovery AFM using a custom programming script. Within the script, the position of each specimen was mapped on the large sample stage, which can accommodate up to 52 samples in one run (Figure S3). A 2 × 2 μm scan size was chosen to balance sufficient resolution of domain morphology with a representative sampling area (Figure S4). To enhance AFM height contrast, the films were immersed in ethanol for 15 min to perform a reconstruction treatment, during which the POEGMA phase was selectively removed. Because ethanol does not dissolve PS, the underlying domain morphology remains unchanged, as confirmed by before-and-after comparisons (Figure S5).^26^ For each sample, nine AFM images were collected from distinct regions to provide a comprehensive representation of morphology, avoiding bias from a single localized image (Figure 3b). Within each batch (36 samples for this work), AFM acquisition is fully automated; however, manual intervention is required between batches for sample exchange. Using this workflow, 2340 AFM images were collected from 260 SPB samples over a total acquisition time of approximately 7 days. The complete AFM data set is available via GitHub (https://github.com/MaResearchLab/supramolecular-polymer-blends-ML). Representative AFM images of SPB thin films with different components, molecular weights, and blending ratios are shown in Figures 3c and S6–S10.

**Figure 3:**
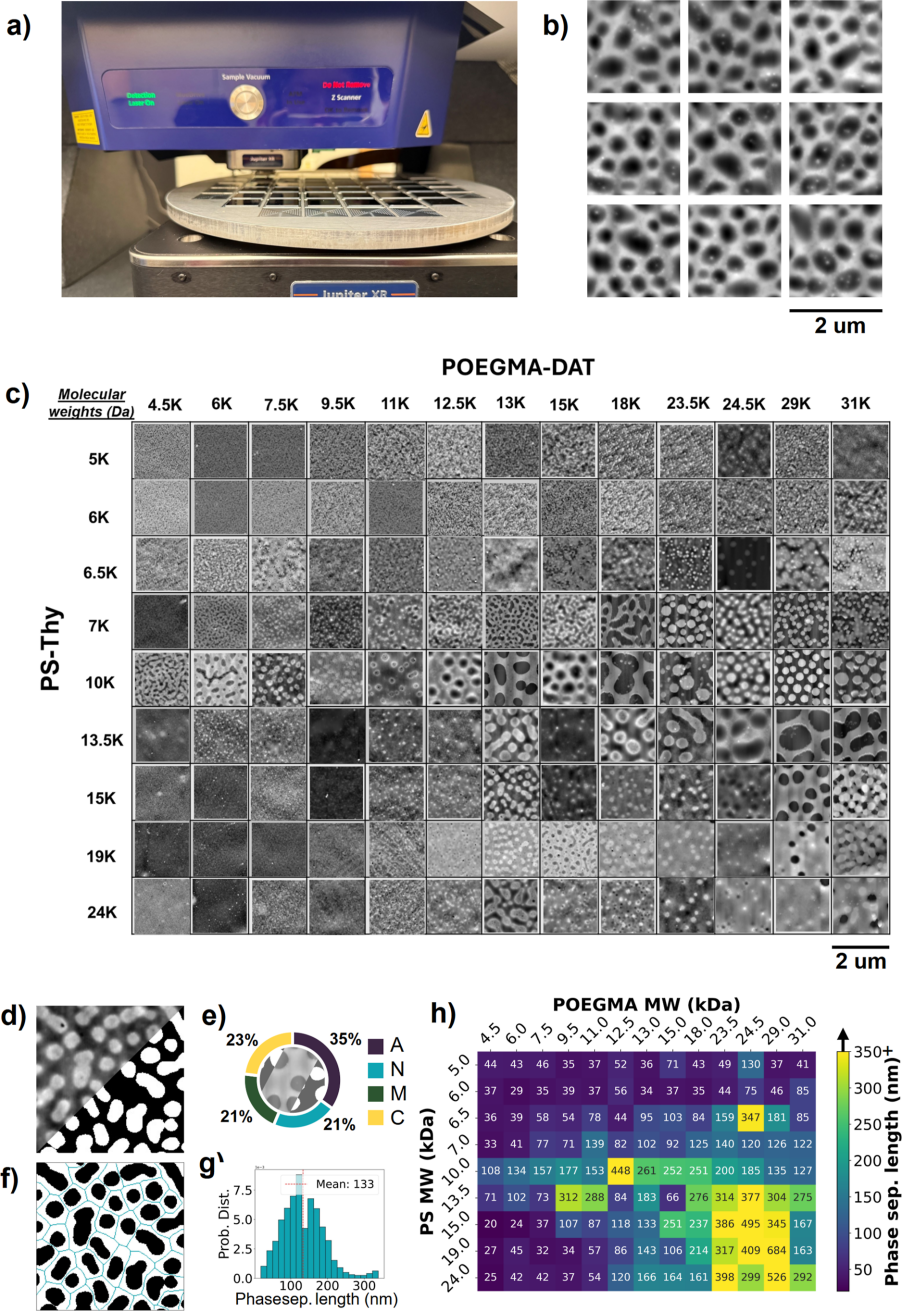
High-throughput phase mapping and data analysis to extract average phase separation size (a) setup of the automated AFM system. (b) Representative set of nine different AFM images collected from a single sample. (c) AFM height image of POEGMA-*sb*-PS after reconstruction in ethanol (scan size: 2 × 2 μm). (d) Representative AFM height images before and after binarization. (e) Image binarization methods, with parameters adjusted on a per-sample basis to achieve the most accurate segmentation. Legend letters indicate Adaptive, Niblack, Manual, and Combination (f) domain spacing extraction based on Voronoi ridge distances between phases and (g) the distribution. (h) Summary of mean domain spacings for POEGMA-*sb*-PS with varying molecular weights.

The acquired AFM images were subsequently binarized and analyzed to quantify the domain spacing. To ensure the reliability of quantitative analysis, only images exhibiting clear and continuous phase-separated domains were included. Specifically, selected images were required to show (i) sufficient phase contrast between domains, (ii) spatially continuous features across the field of view, and (iii) domain sizes significantly larger than the background noise level. Images lacking these characteristics, such as those dominated by surface roughness or weak contrast without well-defined domains, were excluded from the quantitative analysis. Accordingly, a subset of AFM images of POEGMA/PS SPBs was selected for analysis, which includes one representative image within 9 images per sample. To enhance the contrast, images were segmented into majority and minority phases using a combination of adaptive binarization,^27^ Niblack binarization,^28^ and manual segmentation,^29^ as illustrated in Figure 3d,e. Image segmentation remains the most significant bottleneck for high-throughput image analysis, as it is a semimanual step, and the workflow is therefore not fully automated end-to-end. Consequently, the consistency of the segmentation directly affects the quality of the extracted labels used for the machine learning model training. Accurately segmenting our 117-image data set required approximately 5 days. Because the AFM images span a wide range of contrast levels, spatial scales, and morphological features, it is difficult to define a single fixed threshold value that reliably segments all images (examples shown in Figure S11). Instead, threshold values were determined through an iterative adjustment process using a custom segmentation widget (Figure S12). This tool allows rapid tuning of preprocessing and binarization parameters (e.g., filtering and threshold values) while visually inspecting the segmentation results. The threshold for each image was selected such that the resulting binary mask accurately captured the visually identifiable phase-separated domains while avoiding the segmentation of background noise or surface artifacts. The details of the widget are discussed in the Supporting Information. Domain spacings were then extracted from the binarized images by aggregating twice of the Euclidean distance to the nearest minority feature edge from every point along the Voronoi ridge and extracting the mean of the resultant distribution. A representative analyzed AFM image is shown in Figure 3f, where the majority phase is white, and its Voronoi tessellation is shown in blue. The domain spacing distribution of the example image is shown in Figure 3g, with the highlighted distribution mean taken as the characteristic domain spacing. Figure 3h summarizes the domain spacings of SPBs composed of POEGMA-DAT and PS-Thy. Voronoi tessellation was compared against Fourier transform,^30^ edge-to-edge analysis,^31^ and local thickness^32^ analysis to determine an effective metric for phase separation length. Voronoi tessellation outperformed edge-to-edge and Fourier analysis. Local thickness demonstrated comparable performance in both intersample consistency (Figure S13) and model performance (Figure S14), but we focus our discussion on Voronoi tessellation due to faster computation. The image analysis workflow ran smoothly once binary masks were available. The image analysis workflow can be implemented rapidly, with an average throughput of around 17 images per second. Further details for image segmentation and phase separation measurements are described in the Supporting Information.

To achieve a mechanistic understanding of the driving force of phase separation in SPBs, we systematically prepared a series of blends without intermolecular hydrogen-bonding interactions using POEGMA-DAT and PS as a comparison (Figure S15).

For binary polymer blends, when the Flory–Huggins parameter χ is large, enthalpic penalties from unfavorable mixing drive macro-phase separation. This enthalpic contribution originates from the free-energy cost of mixing. However, at low *M*_W_s, the enthalpic penalty is relatively small compared to the configurational entropy of the short chains, resulting in a homogeneous morphology. This explains the absence of phase separation in low-*M*_W_ POEGMA-PS SPBs.

As the molecular weight (*M*_W_) increases, the entropic contribution to mixing decreases, and once the enthalpic penalty dominates, phase separation occurs. In conventional binary polymer blends, this process typically leads to μm-scale domain spacings. In the present supramolecular polymer blend (SPB) system, however, complementary DAT/Thy hydrogen bonding introduces an additional associative interaction that promotes the formation of supramolecular block copolymer-like structures (POEGMA-*sb*-PS), resulting in much finer phase separation on the order of ∼100 nm. Nevertheless, because the two components are connected through reversible hydrogen bonding rather than permanent covalent linkages, the system does not behave like a conventional linear diblock copolymer. In particular, the relatively weak hydrogen-bonding interactions in high-molecular-weight systems, the presence of possible intramolecular associations, and the inevitable fraction of unassociated homopolymers can reduce the effective connectivity between the two polymer segments. As a result, the observed domain spacings remain larger than those typically found in conventional covalently bonded block copolymers, which usually exhibit domain spacings on the order of tens of nanometers. These characteristics highlight that the morphology of SPBs is better understood as polymer blend phase separation modified by supramolecular interactions, rather than as classical block copolymer microphase separation.

At even higher *M*_W_s, domain spacings further increase to 200–400 nm and larger. This is because longer polymer chains diminish the effectiveness of hydrogen bonding in maintaining strong associations, leaving a larger fraction of unassociated homopolymers and thereby enlarging the phase separation distance. The exact fraction of unassociated homopolymers, however, cannot be directly quantified by NMR because the hydrogen-bonding interactions between the DAT and Thy groups are dynamic and reversible in both solution and thin-film states. As a result, the NMR signals represent an averaged state of associated and unassociated species, making it difficult to determine the precise fraction of unbound homopolymers. A similar trend has been reported previously for systems based on ionic interaction.^25^

In addition to molecular weight, the thin-film morphology can also be sensitive to film thickness and processing history. To minimize variability, all samples were prepared by using identical processing conditions. However, maintaining a constant hydrogen-bonding ratio required a fixed molar ratio of homopolymer precursors during spin coating, and thus, film thickness was not strictly controlled, representing a limitation of the current study. The influence of thickness and processing history will be systematically investigated in future work.

Overall, considering the combined effects of molecular weight, supramolecular interactions, and processing-related factors, SPBs consistently exhibit reduced domain spacings compared to nonhydrogen-bonded blends (Figure S15), confirming that hydrogen bonding effectively suppresses macrophase separation between chemically distinct domains.

### Data Curation, ML Training, and Phase Behavior Prediction

The curated SPB data set obtained from high-throughput combinatorial synthesis and characterization was subsequently employed as input for ML model training to probe structure–property relationships and enable morphology prediction and inverse design of SPBs. A range of single-output ML models including Linear, Power, random forest regression (RFR), support vector regression (SVR), extreme gradient boosting regression (XGB), multilayer perceptron (MLP), and Gaussian process regression (GPR) were trained using the phase separation length values obtained from the image analysis (Table S4), where the model takes M_N,POEGMA_ and M_N,PS_ as inputs and predicts the corresponding phase separation length. It should be noted that the ML models were trained using experimentally measured domain spacing values extracted from AFM images, which implicitly capture the net effect of all physical factors in the system, including partial supramolecular association, molecular weight variations, and morphology differences. Therefore, any variability arising from the presence of unassociated homopolymers is inherently incorporated into the training data and does not introduce an additional uncontrolled variable in the model.

Performance of all trained models including *R* ^2^ and root-mean-square error (RMSE) metrics for both training and validation data sets is shown in Figure S16 and Table S4. Among these models, RFR and SVR delivered the best performance metrics. RFR was more effective in inverse design tasks, while the SVR model provided a smooth and continuous response surface, which may better reflect the underlying physical trends in the system. Therefore, both models are discussed in the following analyses. Details of model training, including feature selection and model parameters, are provided in the Supporting Information.

The domain spacings of SPBs predicted by SVR are visualized as a heatmap in Figure 4a. The response surface does reflect qualitative trends in phase separation behavior seen in the AFM image matrix in Figure 4b. Further, the concave shape of the response surface suggests a phase-inversion behavior, where for a fixed *M*_W_ of one component, as the *M*_W_ of the other component increases, domain spacing increases up to a maximum before the minority and majority features flip and the domain spacing decreases. This effect is shown with representative fixed *M*_W_s of PS 10K (blue) and PS 15.2K (green) in Figure 4c with several images along their associated line cuts from the response surface. Figure 4a,b further supports this phase inversion. The highlighted samples have inverse volume fractions and show similar domain spacings.

**Figure 4:**
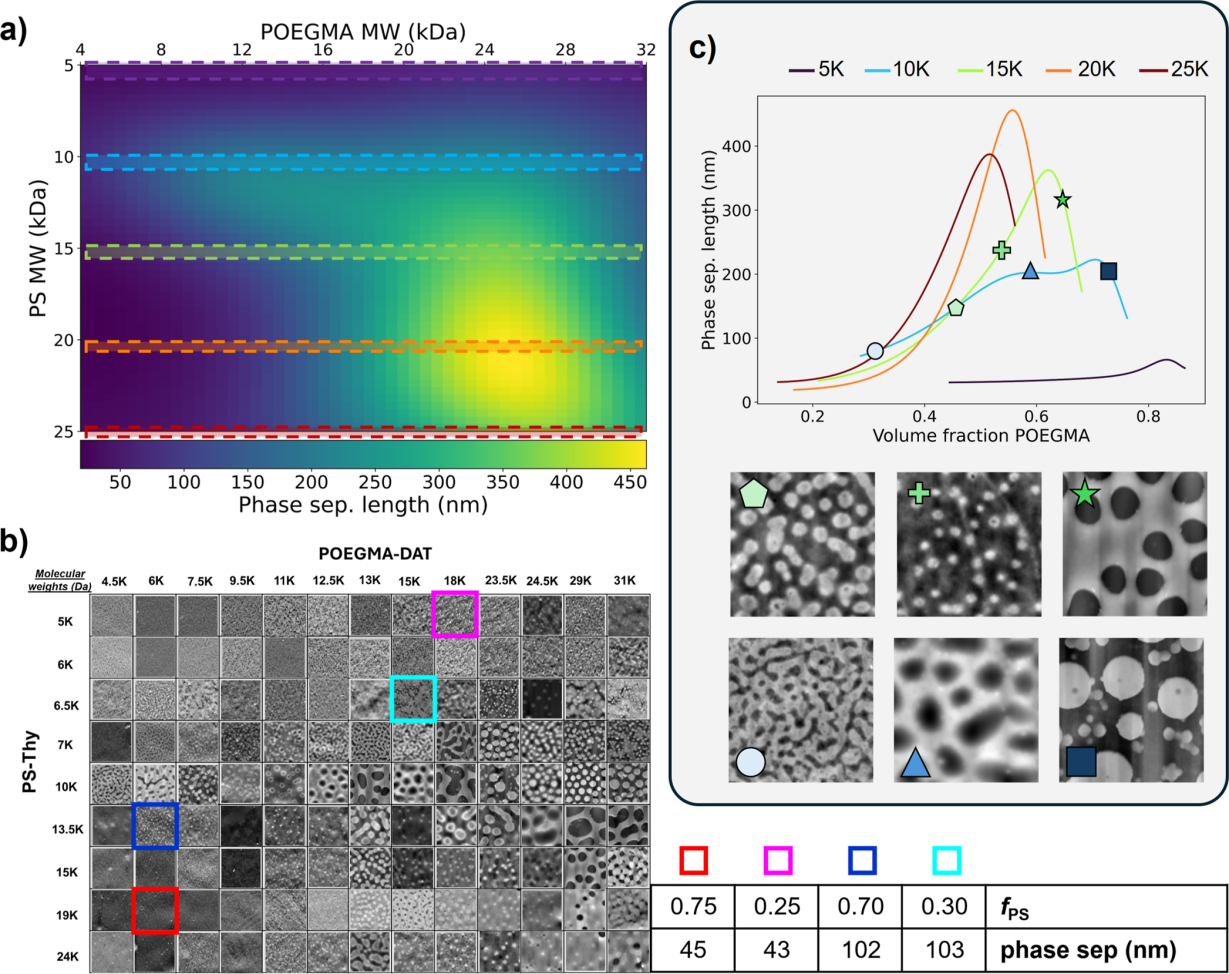
Interfeature spacing and phase inversion of SPB. (a) SVR model predictions for phase separation as a heatmap. (b) AFM image matrix. Colored squares map to the AFM image matrix, and domain spacing values for the highlighted samples are shown in (b), demonstrating phase inversion. (c) 1D plot of line cuts from (a), with selected images along fixed PS *M*_W_, further demonstrating phase inversion.

These trends are not always reflected, in part because the model exhibits a modest coefficient of determination (*R* ^2^) of 0.68 for SVR and 0.69 for RFR. These results indicate that the model captures general trends in phase behavior but does not yet provide high-precision quantitative predictions across the entire parameter space. The parity plot of the RFR model is shown in Figure 5a, and the parity plot of the SVR is shown in Figure S17a, demonstrating that the model underperforms at higher phase separation lengths. While Figure S13 does demonstrate that there is no universally effective single metric for evaluating phase separation in these complex images, it also demonstrates that other approaches also do not adequately address this issue. It is reasonable that the model struggles primarily with an imbalance between the amount of available data at shorter and longer phase separation lengths, as shown by the residuals analysis in Figure S18, where the data density is much higher below the 200 nm phase separation length.

**Figure 5:**
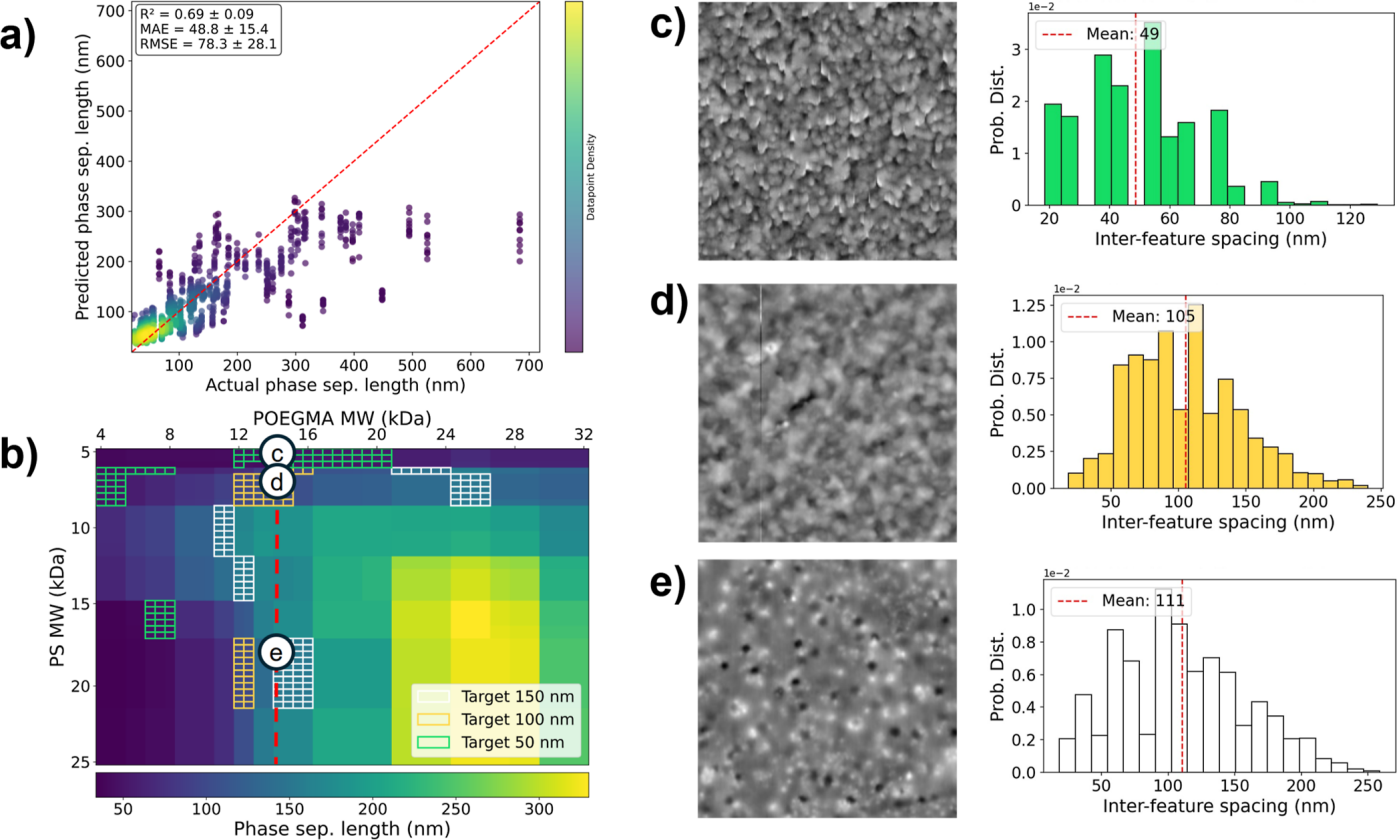
ML-based morphology prediction and inverse design of SPBs. (a) Parity plot of measured versus predicted domain spacings from the optimized RFR model. (b) RFR prediction heatmap highlighting compositions within ±5 nm of selected targets (50, 100, and 150 nm). Experimental validation for inverse design at these targets is shown in (c) 50 nm, (d) 100 nm, and (e) 150 nm. All AFM images are 2 × 2 μm.

We demonstrate a proof-of-concept inverse design capability of the model to develop new SPBs to obtain targeted domain spacings. First, we used both the RFR and SVR models to predict the *M*_W_s of each component needed to synthesize SPBs with domain spacings of 50, 100, and 150 nm. Then, the predicted polymers were synthesized, processed, characterized, and compared to the predicted values. For three domain spacing targets, all feasible regions were highlighted in the predicted heatmap within a tolerance of 5 nm (Figures 5b and S17b). The RFR model was better at this inverse design task and is highlighted for discussion; SVR results are in the Supporting Information. The RFR suggested that POEGMA/PS SPB samples with *M*_W_s of 14.3k–5.0k, 14.3k–6.2k, and 14.3k–18.9k could yield targeted domain spacings. Using RAFT polymerization, we successfully made a new POEGMA with an *M*_W_ of 14.3k and sequentially blended with PS to achieve 14.3–5.0k, 14.3k–6.2k, and 14.3k–18.7k binary blends. The surface morphology for those SPBs was characterized by AFM, and their domain spacing was measured using the same computational workflow previously described (Figure 5c–e). We observed domain spacing means of 48.7, 105.2, and 110.5 nm, respectively, for each of the made and measured samples, in reasonable agreement with the prediction. Absolute discrepancies between measurements and targets were 1.3, 5.2, and 39.5 nm, respectively, or a mean absolute error (MAE) of only 17.3 nm between the predictions and actual values, and an MAE of 10.8 nm between the targets and the samples made (Figure 5c–e). Tabulated results for both the RFR and SVR models are shown in Tables S5 and S6. Unsurprisingly, the model is slightly weaker at inverse design tasks targeting larger phase separation lengths, due to shortcomings in the phase separation metric and image segmentation as discussed previously. In addition, it should also be noted that the current machine learning model is trained on morphologies obtained under specific processing conditions; therefore, the predictions inherently reflect these conditions. Variations in film thickness and processing history can also influence the resulting morphology, and thus, the predictive capability of the model is primarily applicable within the same processing framework.

## Conclusion

In this study, we established an automated, high-throughput approach to supramolecular polymer blend design by integrating modular synthesis, robotic blending, automated AFM imaging and analysis, and ML-guided phase behavior prediction. From only 33 homopolymer precursors, 260 SPBs were fabricated and fully characterized in weeks rather than years, yielding 2340 high-quality morphology images. These data enabled the development of predictive ML models capable of mapping molecular features to domain spacings and, importantly, performing a proof-of-concept inverse design with experimental validation. Our findings underscore the transformative potential of combining automation and ML in polymer research: enabling rapid data set generation, improving predictive accuracy, and expanding the accessible design space of SPBs. Beyond the specific system studied here, this workflow provides a generalizable strategy that can be extended to other supramolecular interactions, polymer architectures, and multimodal characterization techniques. The automation levels for each step are summarized in Table 1. By integrating synthesis, characterization, and ML-driven prediction, this work advances the rational design of functional supramolecular polymer materials and paves the way for an accelerated materials discovery.

**Table 1:** Summary of the Workflow Used in This Study, Indicating the Automation Level of Each Stage from Material Synthesis to Machine Learning Prediction

step	method	automation level	approximate time
Homopolymer synthesis	Modular synthesis	Manual	∼3 months
SPB fabrication	Liquid-handling robot	Automated	1 day
Thin-film preparation	Spin coating	Manual	2–3 days
AFM acquisition	Automated AFM workflow	Automated per 36-sample batch	∼7 days
Image segmentation	Widget tuning	Semiautomated	∼5 days
Feature extraction	Voronoi spacing analysis	Automated	1 day
ML prediction	RFR/SVR models	Automated	1 day

## Supplementary Material



## Notes

Materials and methods; modular synthesis of DAT/Thy-functional homopolymer precursors; automated and high-throughput fabrication of supramolecular block copolymers; automated atomic force microscopy (AFM); AFM image binarization and domain spacing extraction; morphology explanation of other SPBs; machine learning-guided morphology prediction and inverse SPB design; and additional results (PDF)
