# Supplementary material for: Automated and High-Throughput Phase Separation Control for Supramolecular Polymer Blends Enabled by Machine Learning
Source: JACS Au. 2026 May 29;6(6):3153–63. doi: 10.1021/jacsau.6c00041 (PMC13291874; doi:10.1021/jacsau.6c00041)
Supplement: Supplementary file 1 [file au6c00041_si_001.pdf]

Supporting Information

Automated and High-Throughput Phase Separation Control for Supramolecular Polymer Blends Enabled by Machine Learning

Yunfei Wang,[1,2] Daniel Struble,[1] Saroj Upreti,[1] Zongliang Xie,[3] Ka Hung Chan,[2] Yi Liu,[3] Chenhui Zhu,[2] Paul Ashby,[3] Wenjie Xia[4], Derek Patton[1], Boran Ma,[1,*] Xiaodan Gu[1,*]

1 School of Polymer Science and Engineering, Center for Optoelectronic Materials and Devices, the University of Southern Mississippi, Hattiesburg, MS 39406, USA

2 Advanced Light Source, Lawrence Berkeley National Laboratory, Berkeley, CA 94720, USA

3 The Molecular Foundry, Lawrence Berkeley National Laboratory, Berkeley, CA 94720, USA

4 Department of Aerospace Engineering, Iowa State University, Ames, IA 50011, USA

The PDF file includes:

Materials and Methods

Supplementary Text

Figs. S1 to S18

Tables S1 to S6

References

The Supplementary Materials includes:

1. Materials and methods

2. Modular synthesis of DAT/Thy-functional homopolymer precursors

3. Automated and high-throughput fabrication of supramolecular block copolymers

4. Automated atomic force microscopy (AFM)

5. AFM image binarization and domain spacing extraction

6. Morphology explanation of other SPBs

7. Machine learning-guided morphology prediction and inverse SPB design

1. Modular synthesis of DAT/Thy-functional homopolymer precursors

__Overview of the synthesis of DAT-alkyne and Thy-alkyne__

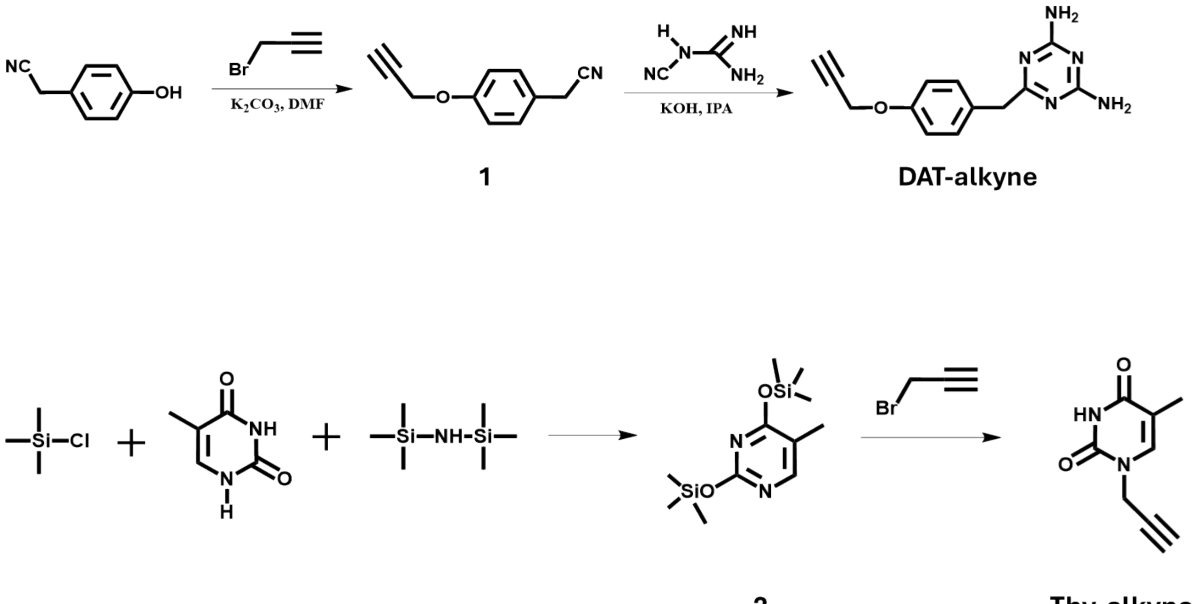

Figure S1a. Synthetic route of DAT-alkyne and Thy-alkyne

__{4-[(prop-2-yn-1-yl)oxy]phenyl}acetonitrile (**1**)__

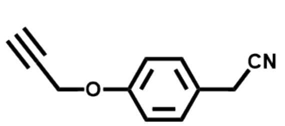

1

Synthesis of Compound 1: Compound 1 was synthesized according to a previously reported precedure.[1] A round-bottom flask equipped with a magnetic stir bar was charged with 4-hydroxyphenylacetonitrile (1.57 g, 11.8 mmol), potassium carbonate (3.26 g, 23.6 mmol), propargyl bromide (3.51 g, 23.6 mmol) and DMF (40 ml). The mixture was stirred at 70 °C for 24 hours. Upon completion of the reaction, the mixture was poured into cold water (400 mL). The organic layer was extracted with chloroform, washed once with

brine and twice with H_2O, dried over Na_2SO_4, and concentrated under reduced pressure. The crude product was purified by flash chromatography on silica gel using DCM as the eluent to afford **Compound 1** as a yellow solid (2.87 g, 70% yield).

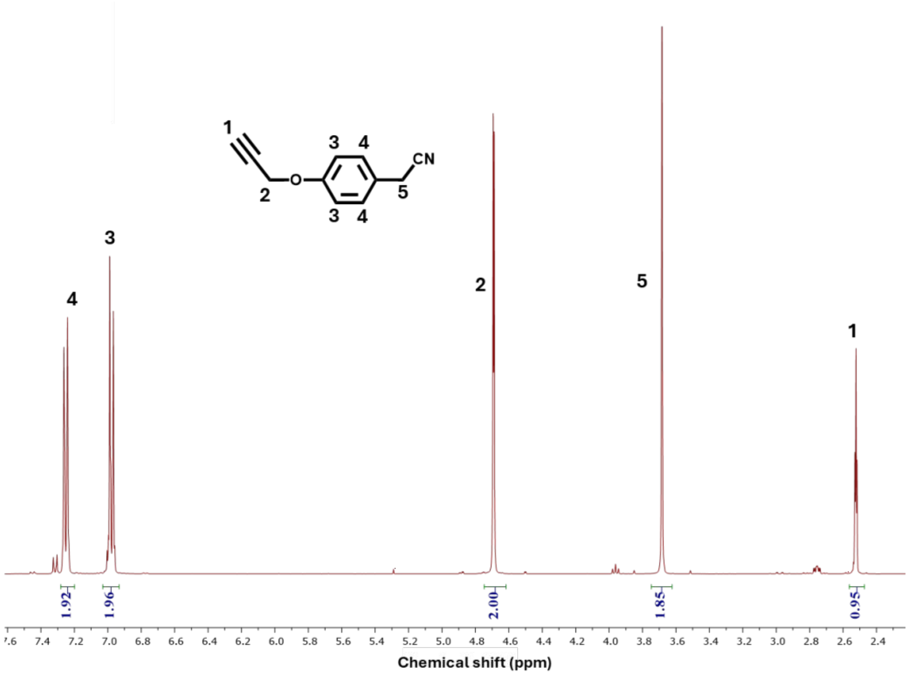

Figure S1b. ^{1}H NMR spectrum of **compound 1** in $CDCl_3$

<u>6-({4-[(prop-2-yn-1-yl)oxy]phenyl}methyl)-1,3,5-triazine-2,4-diamine (**DAT-Alkyne**)</u>

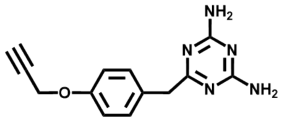

DAT-alkyne

Synthesis of DAT-Alkyne: DAT-alkyne was synthesized according to a previously reported precedure.[1] The synthesis was carried out under a dry nitrogen atmosphere. All glassware was heated under vacuum and flushed with nitrogen several times before chemicals were weighed in. A three-neck round-bottom flask equipped with a magnetic stir bar was charged with **compound 1** (1.00 g, 5.8 mmol), dicyandiamide (0.98

g, 11.6 mmol), potassium hydroxide (0.07 g, 1.2 mmol) and anhydrous isopropanol. The mixture was stirred at 85 °C for 24 hours. The crude product was purified by chromatography on silica gel using chloroform, followed by chloroform/methanol (8:1), to afford DAT-alkyne as a light yellow solid (2.87 g, 85% yield). The NMR spectrum of DAT-alkyne is shown below.

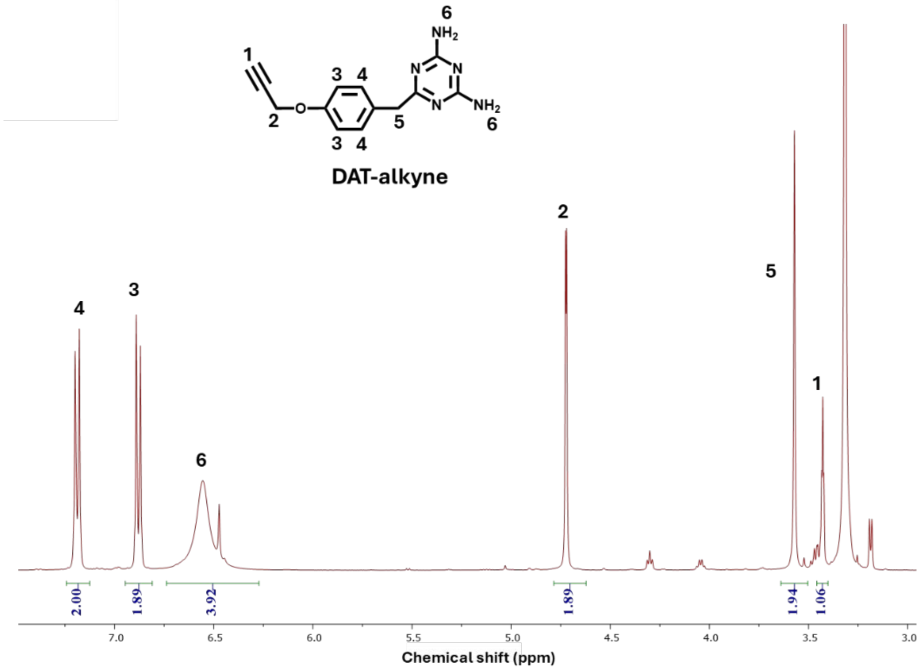

Figure S1c. ^{1}H NMR spectrum of **DAT-alkyne** in $CDCl_3$

<u>5-methyl-1-(prop-2-yn-1-yl)pyrimidine-2,4(1*H*,3*H*)-dione</u> (**Thy-alkyne**)

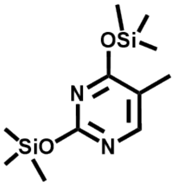

2

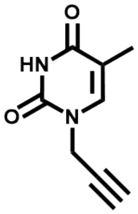

Thy-alkyne

Compound 2 and Thy-Alkyne: Compound 2 and Thy-Alkyne were synthesized according to a previously reported procedure.[2] A round-bottom flask equipped with a magnetic stir bar was charged with chlorotrimethylsilane (0.66 g, 7.1 mmol), thymine (3.84 g, 30.5 mmol) and hexamethyldisilazane (5.89 g, 36.5 mmol). The mixture was reflux at 130 °C for 24 hours. Upon completion, the excess hexamethyldisilazane was removed under reduced pressure to obtain crude **compound 2**. Without further purification, propargyl bromine (3.35 g, 28.2 mmol) was added to the mixture, which was then stirred at room temperature for 9 days. After completion of the reaction, H_2O was added. The organic layer was extracted with chloroform (5 times in volume). The combined organic extracts were washed twice with H_2O, dried with Na_2SO_4 , and concentrated under reduced pressure to yield **Thy-alkyne** as a light yellow solid (2.20 g, 47% yield). The NMR spectrum of Thy-alkyne is shown below.

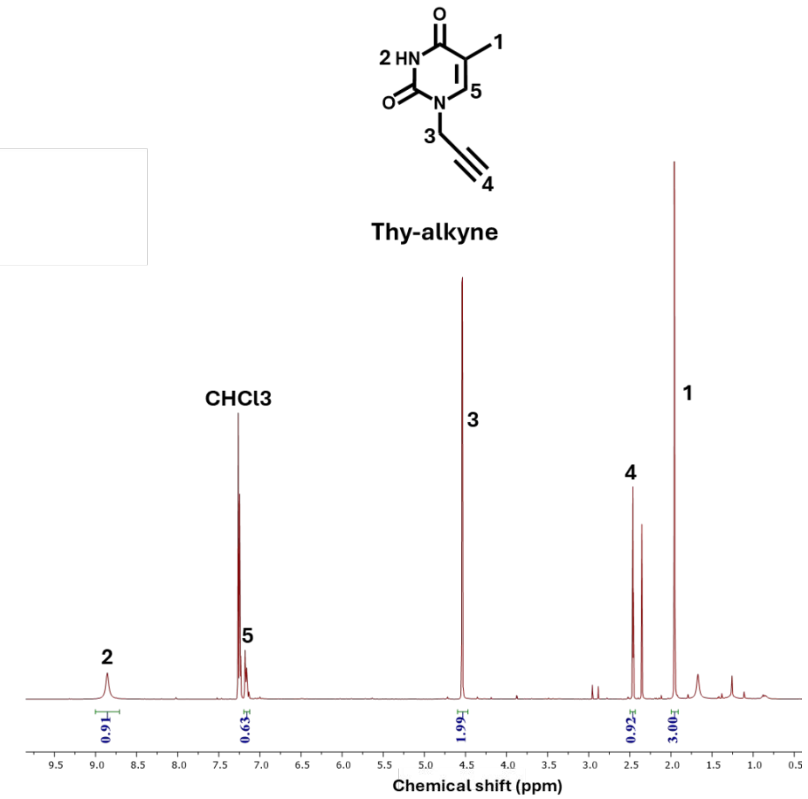

Figure S1d. ^{1}H NMR spectrum of **Thy-alkyne** in $CDCl_3$

Azide-functional chain transfer agent (CTA-N3)

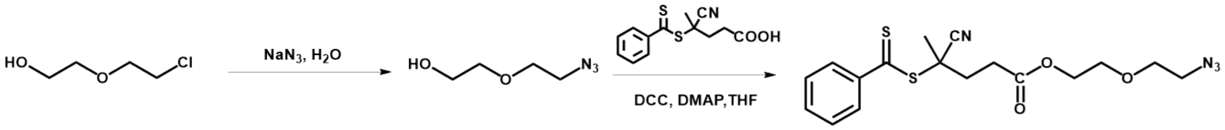

3 **CTA-N3**

Figure S1e. Synthetic route of azide-functional CTA (**CTA-N3)**.

2-(2-azidoethoxy)ethan-1-ol (3)

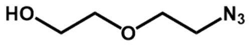

3

Compound 3: Compound 3 was synthesized according to a previously reported procedure.[3] A round-bottom flask equipped with a magnetic stir bar was charged with 2-(2-chloro-ethoxy)-ethanol (7.50 g, 60 mmol), sodium azide (7.85 g, 120 mmol) and H_2O (25 ml). The mixture was stirred at 90 °C for 16h. Upon completion, the reaction mixture was cooled to room temperature and extracted with DCM (6 x 25 ml). The combined organic layers were dried over Na_2SO_4, and the solvents were removed under reduced pressure to afford **compound 3** as a colorless liquid (7.03 g, 89% yield).

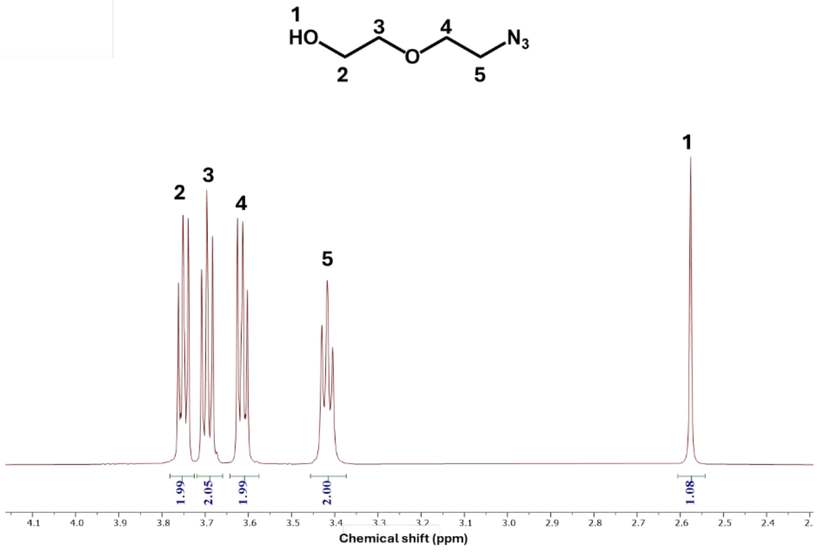

Figure S1f. ^{1}H NMR spectrum of **Compound 3** in $CDCl_3$

2-(2-azidoethoxy)ethyl 4-[(benzenecarbothioyl)sulfanyl]-4-cyanopentanoate (**CTA-N$_3$**)

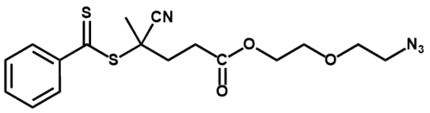

CTA-N3

CTA-N$_3$: CTA-N_3 was synthesized according to a previously reported procedure.[3] A round-bottom flask equipped with a magnetic stir bar was charged with CPADB (2.00 g, 7.2 mmol) and anhydrous THF (100 ml). The solution was cooled to 0 °C under a nitrogen atmosphere. After 15 min, DCC (1.63 g, 7.9 mmol) was added to the reaction mixture, followed by adding DMAP (0.09 g, 0.7 mmol) in the mixture. After an additional 15 min, compound 3 (1.13 ml, 8.6 mmol) was added. The mixture was stirred for 30 min in an ice bath and further stirred overnight at room temperature. Upon completion, the white dicyclohexyl urea (DCU) precipitate was removed by filtration, and the solvent was evaporated under reduced pressure. The crude product was purified by column chromatography on silica gel, eluting with hexane:ethyl acetate (5:1), afford **CTA-N$_3$** as a pink oil (1.51 g, 53 % yield). Its NMR spectrum is shown below.

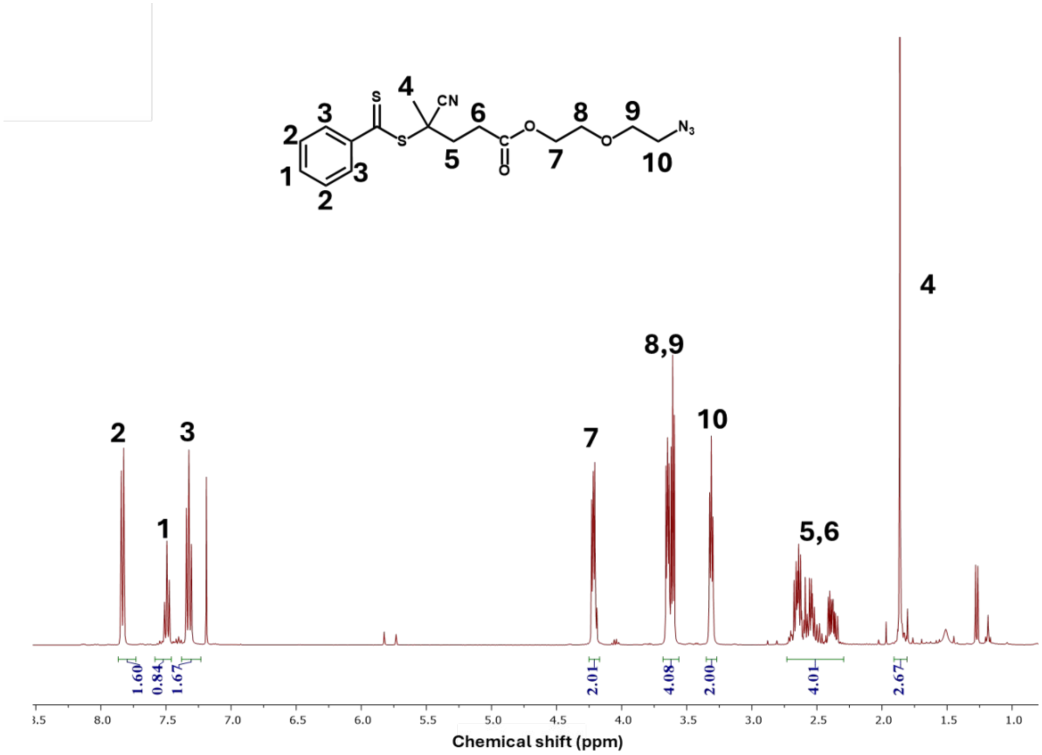

Figure S1g. ^{1}H NMR spectrum of **CTA-N_3** in $CDCl_3$

<u>DAT/Thy-CTA for RAFT polymerization using CuAAc click reaction</u>

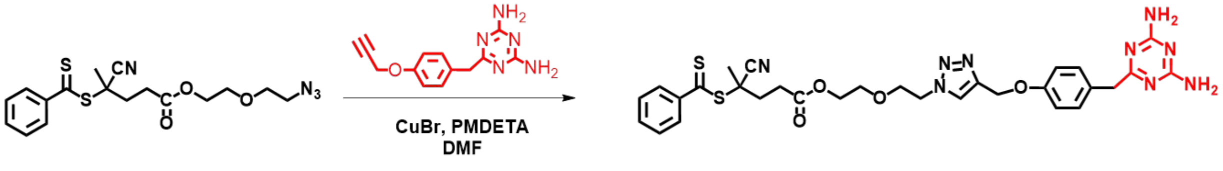

CTA-N_3

CTA-DAT

Figure S1h. Synthetic route of CTA-DAT.

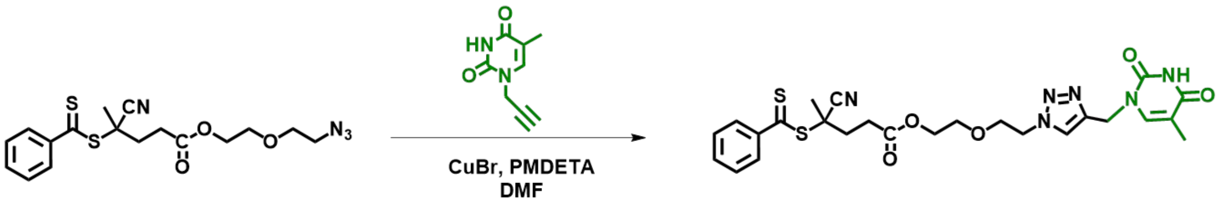

CTA-N3 CTA-Thy

Figure S1i. Synthetic route of CTA-Thy.

<u>**General procedure for CuAAc click reaction of DAT/Thy-CTA**</u>

A round-bottom flask equipped with a magnetic stir bar was charged with CTA-N_3, DAT-alkyne (or Thy-alkyne), PMDETA and anhydrous DMF. The solution was degassed by bubbling dry nitrogen gas for 15 min, after which CuBr was added into the flask under a nitrogen atmosphere. The mixture was stirred at 45 °C for 20 hours and then cooled to room temperature. The DMF was removed under reduced pressure. The crude product was purified by column chromatography on aluminum oxide (neutral) to remove the copper catalyst, followed by column chromatography on silica gel using chloroform/methanol (8:1) as the eluent, to afford DAT-CTA (or Thy-CTA).

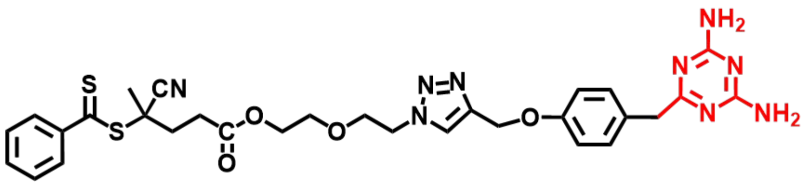

CTA-DAT

CTA-DAT: CTA-N_3 (2.37 g, 8.5 mmol), DAT-alkyne (2.17 g, 8.5 mmol), PMDETA (0.15 g, 0.85 mmol), and CuBr (0.12 g, 0.85 mmol) were dissolved in anhydrous DMF (6 ml). The reaction afforded the project as a pink oil (1.42g, 25% yield).

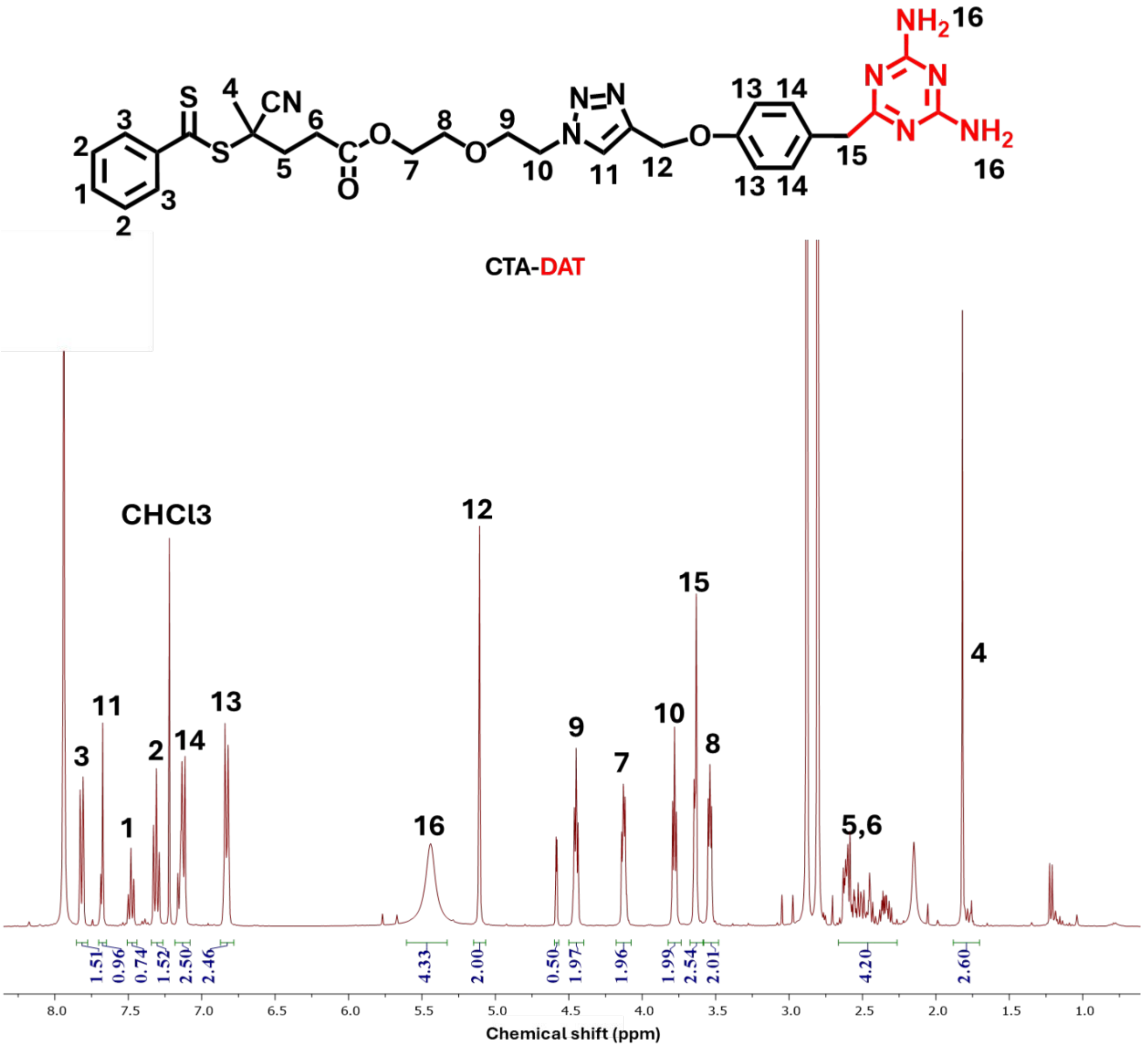

Figure S1j. ^{1}H NMR spectrum of **CTA-DAT** in $CDCl_3$

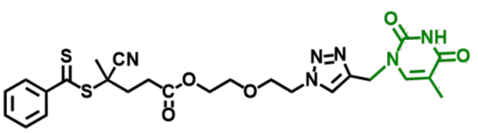

CTA-Thy

CTA-Thy: CTA-N_3 (1.50 g, 5.3 mmol), Thy-alkyne (0.88 g, 5.3 mmol), PMDETA (0.09 g, 0.53 mmol), and CuBr (0.08 g, 0.53 mmol) were dissolved in anhydrous DMF (6 ml). The reaction afforded the product as a pink oil (0.46 g, 20% yield)

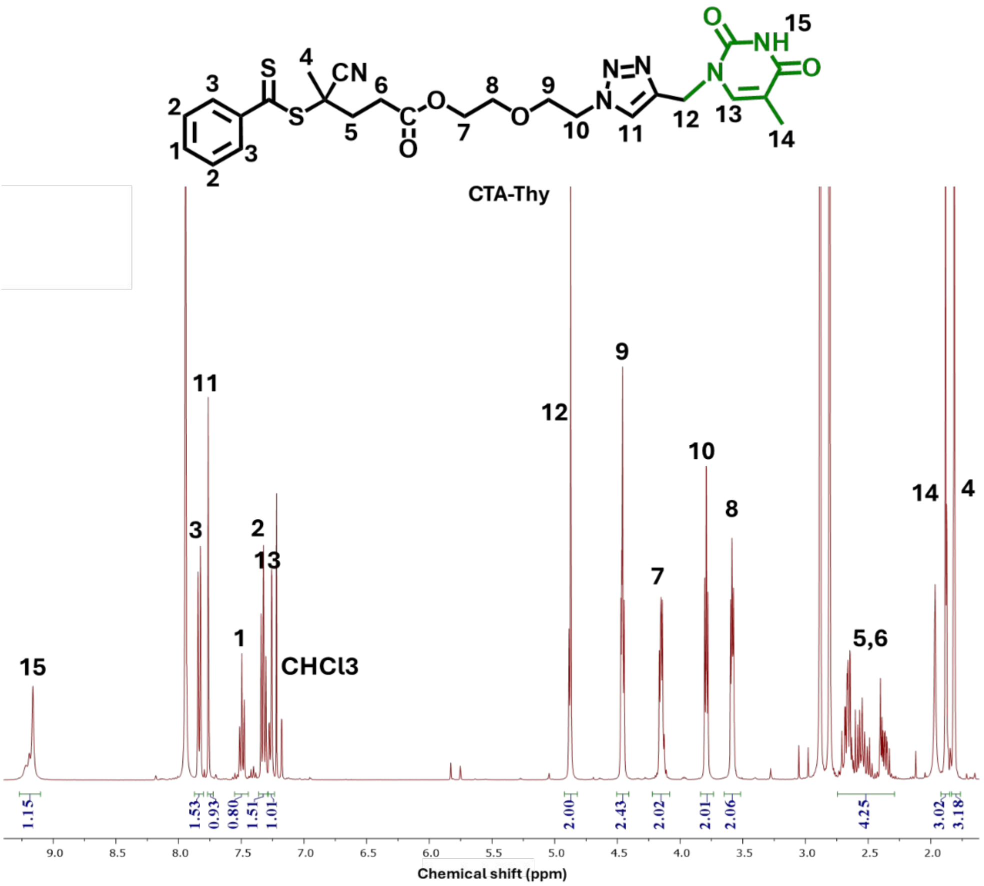

Figure S1k. ^{1}H NMR spectrum of **CTA-Thy** in $CDCl_3$

Synthesis of DAT/Thy-homopolymers

General procedure for RAFT polymerization of DAT/Thy-polymers using 'grafting-from' method

POEGMA-DAT and PS-Thy were prepared by RAFT polymerization using 'grafting-from' method, in which polymers were polymerized using CTA bearing DAT or Thy groups. Typically, a round-bottom flask equipped with a magnetic stir bar was charged with CTA-DAT (or CTA-Thy), the monomer, AIBN and solvent. The solution was degassed by bubbling dry nitrogen gas for 15 min. The polymerization time and temperature varied depending on the specific polymer. Upon completion, the reaction was quenched by immersing the flask in liquid nitrogen. After warming to room temperature, the reaction mixture was precipitated into a 10-fold excess of precipitation solvent to yield homopolymers.

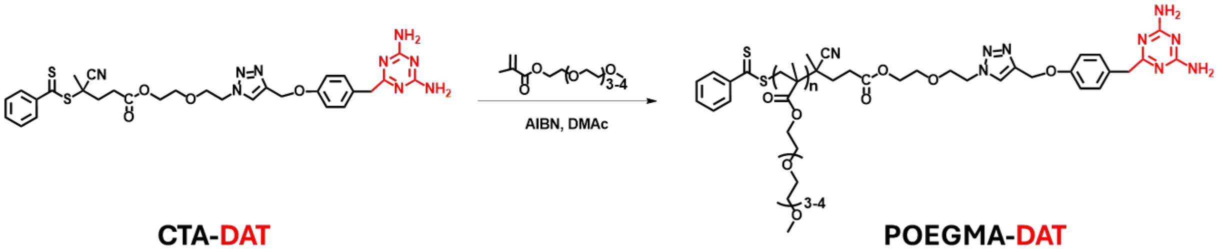

Figure S1l. Synthetic route of **POEGMA-DAT**.

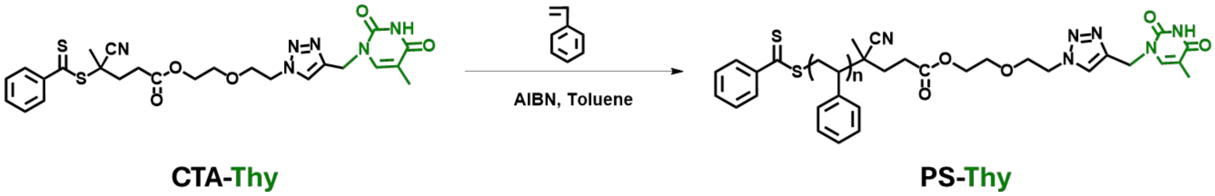

Figure S1m. Synthetic route of **PS-Thy**.

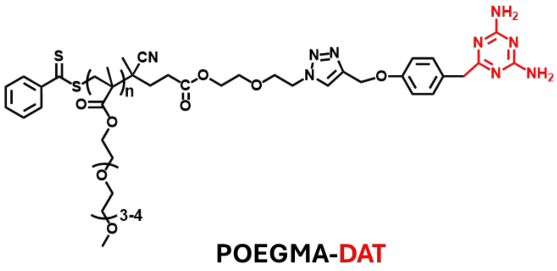

POEGMA-DAT: CTA-DAT (3.24 g, 5 mmol), $OEGMA_{300}$ (75.00 g, 250 mmol), AIBN (273.68 mg, 1.7 mmol) and anhydrous DMAc (20 ml) were combined. The reaction was carried out at 70 °C for 90 min. The product was precipitated in hexane to afford POEGMA-DAT as a pink gel.

To synthesize POEGMA-DAT with different molecular weights, the polymerization time was kept constant while the molar ratio of [OEGMA]:[CTA-DAT]:[AIBN] = target DP:1:0.3 was adjusted to achieve different

target DP. Conventional SEC measurements are not suitable for molecular weight calculation of branched polymers.[4] Therefore, ^{1}H NMR in $CDCl_3$ was used to evaluate MW of POEGMA-DAT comparing the -CH_2- proton resonance in CTA (peak a) and -CH_3 proton resonance in the repeating units (peak b). SEC-MALLS in DMF was used to evaluate the molecular weight distribution ***(Đ)***. The molecular parameters of POEGMA-DAT are summarized in **Table S1**.

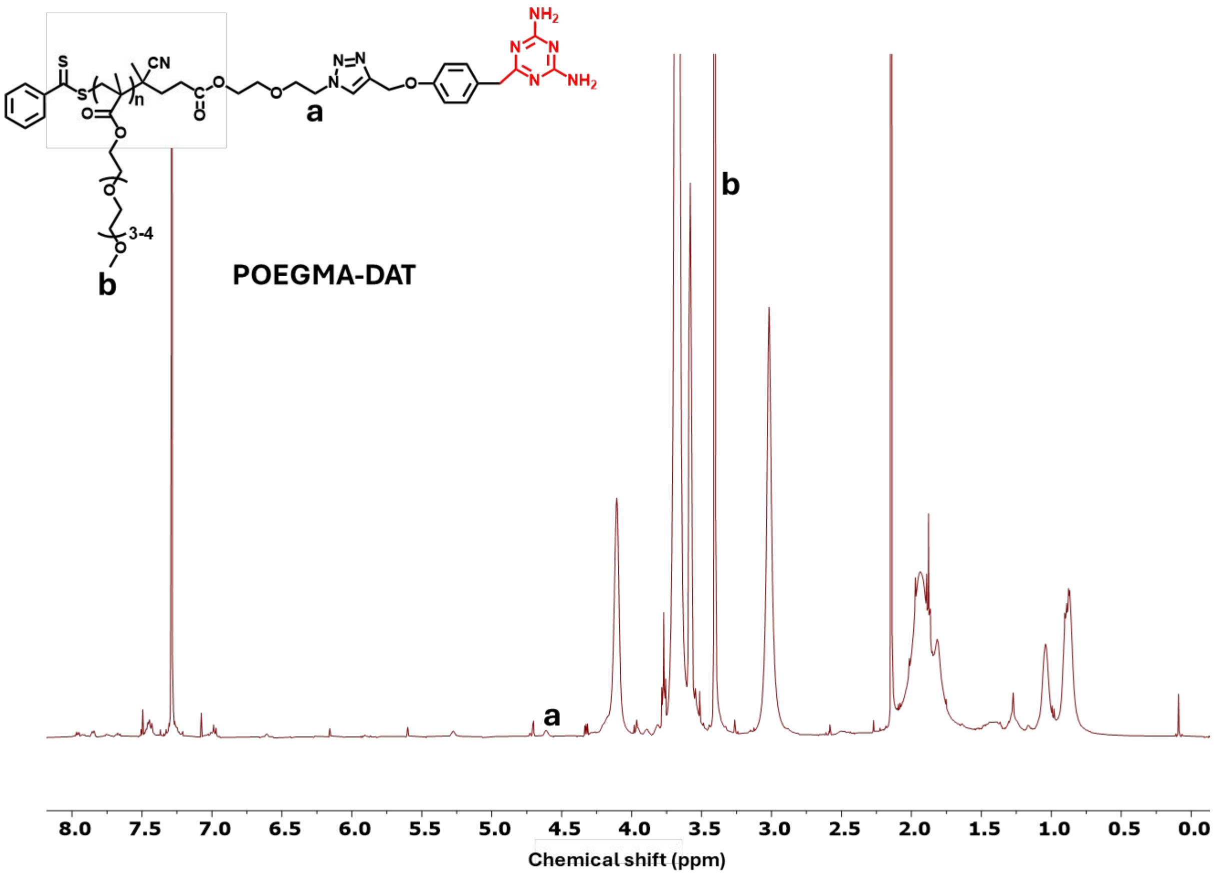

Figure S1n. ^{1}H NMR spectrum of **POEGMA-DAT** in $CDCl_3$

Table S1. Structure properties of the POEGMA-DAT.

Polymers	M_n (kDa)[a]	***Đ***[b]

POEGMA-DAT-4.5k	4.5	1.16
POEGMA-DAT-6k	5.8	1.12
POEGMA-DAT-7.5k	7.6	1.18
POEGMA-DAT-9.5k	9.4	1.22
POEGMA-DAT-11k	11.2	1.19
POEGMA-DAT-12.5k	12.5	1.20
POEGMA-DAT-13k	13.1	1.16
POEGMA-DAT-14k	14.2	1.16
POEGMA-DAT-15k	14.8	1.23
POEGMA-DAT-18k	17.7	1.16
POEGMA-DAT-23.5k	23.8	1.20
POEGMA-DAT-24.5k	24.3	1.39
POEGMA-DAT-29k	28.7	1.24
POEGMA-DAT-31k	30.6	1.40
[a] Determined by ^{1}H NMR. [b] Determined by SEC-MALLS		

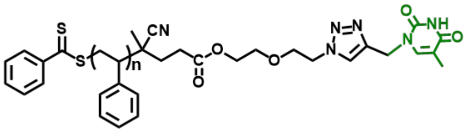

PS-Thy

PS-Thy: CTA-Thy (0.60 g, 1.4 mmol), styrene (26.04 g, 250 mmol), AIBN (76.02 mg, 0.5 mmol) and anhydrous toluene (250 ml) were combined. The reaction was carried out at 75 °C for 18 hours, and the product was precipitated in methane to afford PS-Thy as a pink solid.

To synthesize PS-Thy with different molecular weights, the polymerization time was kept constant while the molar ratio of [styrene]:[CTA-Thy]:[AIBN] = target DP:1:0.3 was adjusted to achieve different target DPs. ^{1}H NMR in $CDCl_3$ was used to confirm the successful synthesis of PS-Thy. SEC-MALLS in DMF

was used to evaluate the molecular weights and molecular weight distribution ***Đ)***, which are summarized in **Table S2**.

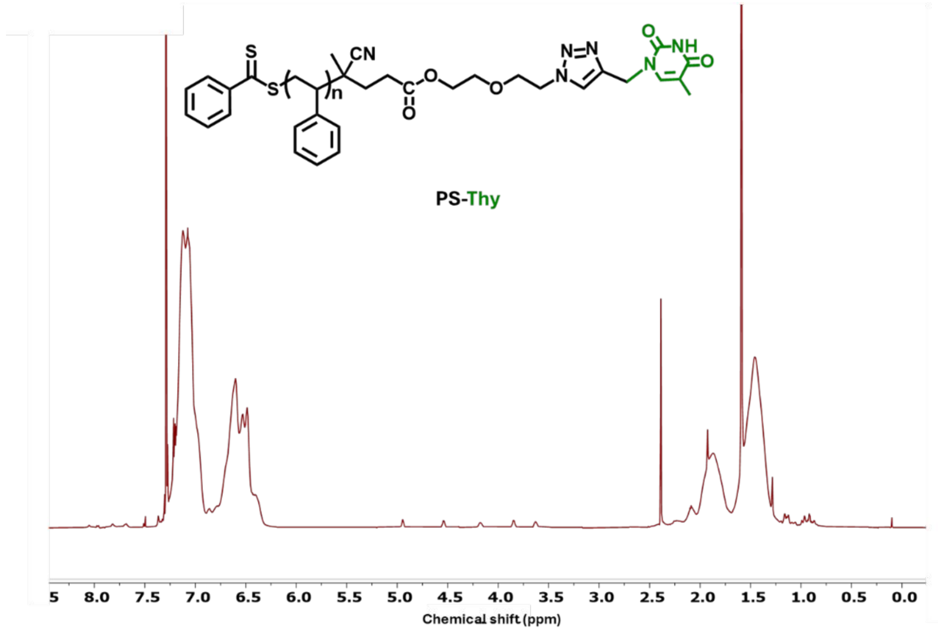

Figure S1o. ^{1}H NMR spectrum of **PS-Thy** in $CDCl_3$

Table S2. Structure properties of the PS-Thy.

Polymers	M_n (kDa)[a]	$Đ$[b]
PS-Thy-3k	3.0	1.09
PS-Thy-5k	5.0	1.08
PS-Thy-6k	6.0	1.07
PS-Thy-6.5k	6.2	1.09
PS-Thy-7k	7.0	1.13
PS-Thy-10k	10.0	1.16
PS-Thy-13.5k	13.6	1.19
PS-Thy-15k	15.2	1.12

PS-Thy-19k	18.7	1.12
PS-Thy-24k	24.0	1.12
[a] Determined by ^{1}H NMR. [b] Determined by SEC-MALLS		

General procedure for DAT/Thy-polymers using ‘grafting-to’ method

PMMA-DAT, PMMA-Thy, PEG-DAT were also synthesized using ‘grafting-to’ method. In which, DAT/Thy functional groups were further conjugated after synthesis of azide functional polymers. The ‘grafting-to’ method was processed by following step: 1) synthesis of azide-functional polymers, 2) click reaction between azide-functional polymers and alkyne-DAT/Thy to generate DAT/Thy-polymers.

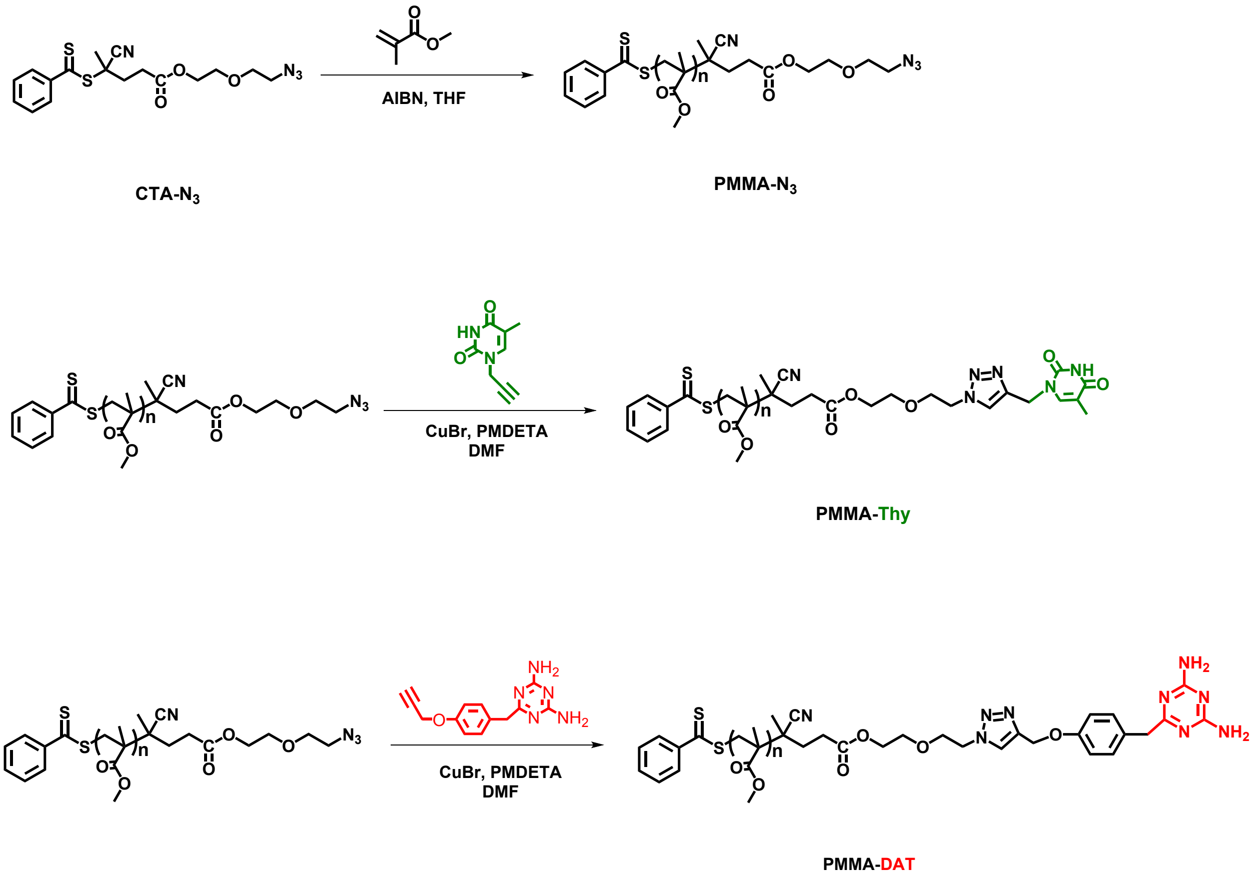

Figure S1p. Synthetic route of **PMMA-DAT/Thy**.

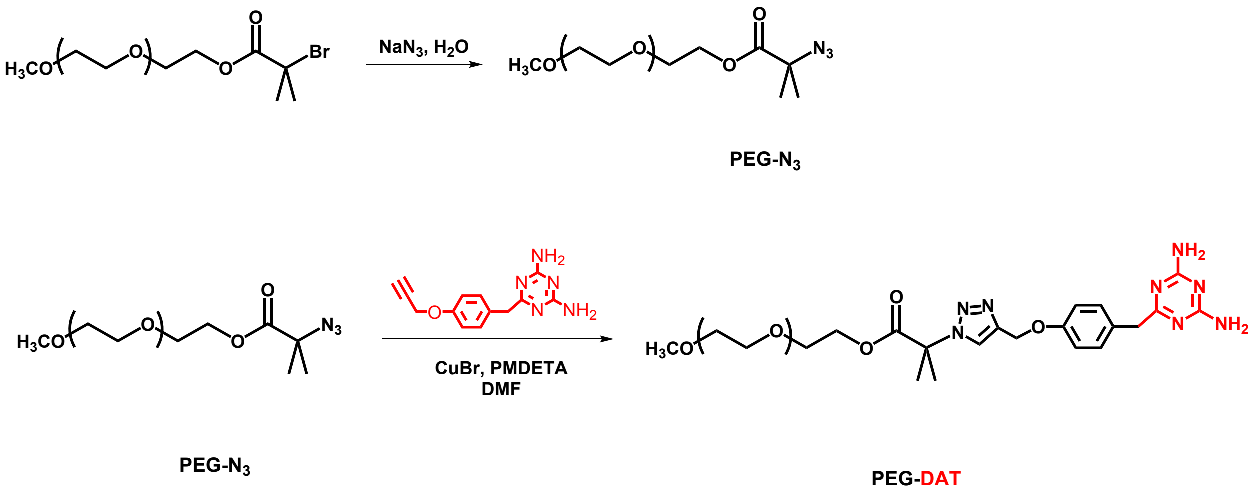

Figure S1q. Synthetic route of **PEG-DAT**.

RAFT polymerization of azide-functional PMMA ($PMMA-N_3$)

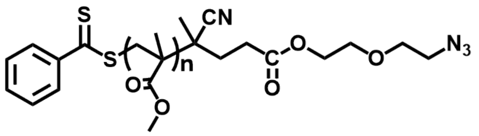

$PMMA-N_3$

A round-bottom flask equipped with a magnetic stir bar was charged with $CTA-N_3$ (0.53 g, 1.35 mmol), MMA (15.02 g, 150 mmol), AIBN (74.64 mg, 0.45 mmol) and THF (150 mmol). The solution was degassed by bubbling dry nitrogen gas for 15 min, followed by heating at 75 °C for 8 h. Upon the completion of reaction, the round-bottom flask was immersed in liquid nitrogen to quench the reaction. After warming to room temperature, the reaction mixture was precipitated in 10-fold excess of hexane and re-dissolve in THF twice to yield PMMA-N3 as pink solid.

To synthesize $PMMA-N_3$ with different molecular weights, the polymerization time was kept constant but the molar ratio of [MMA]:[$CTA-N_3$]:[AIBN] = target DP:1:0.3 was tune to different target DP. ^{1}H NMR in

$CDCl_3$ was used to evaluate MW of PMMA-N_3. SEC-MALLS in THF was used to evaluate the molecular weight distribution ***(Đ)***. The molecular parameters of PMMA-N_3 are summarized in **Table S3**.

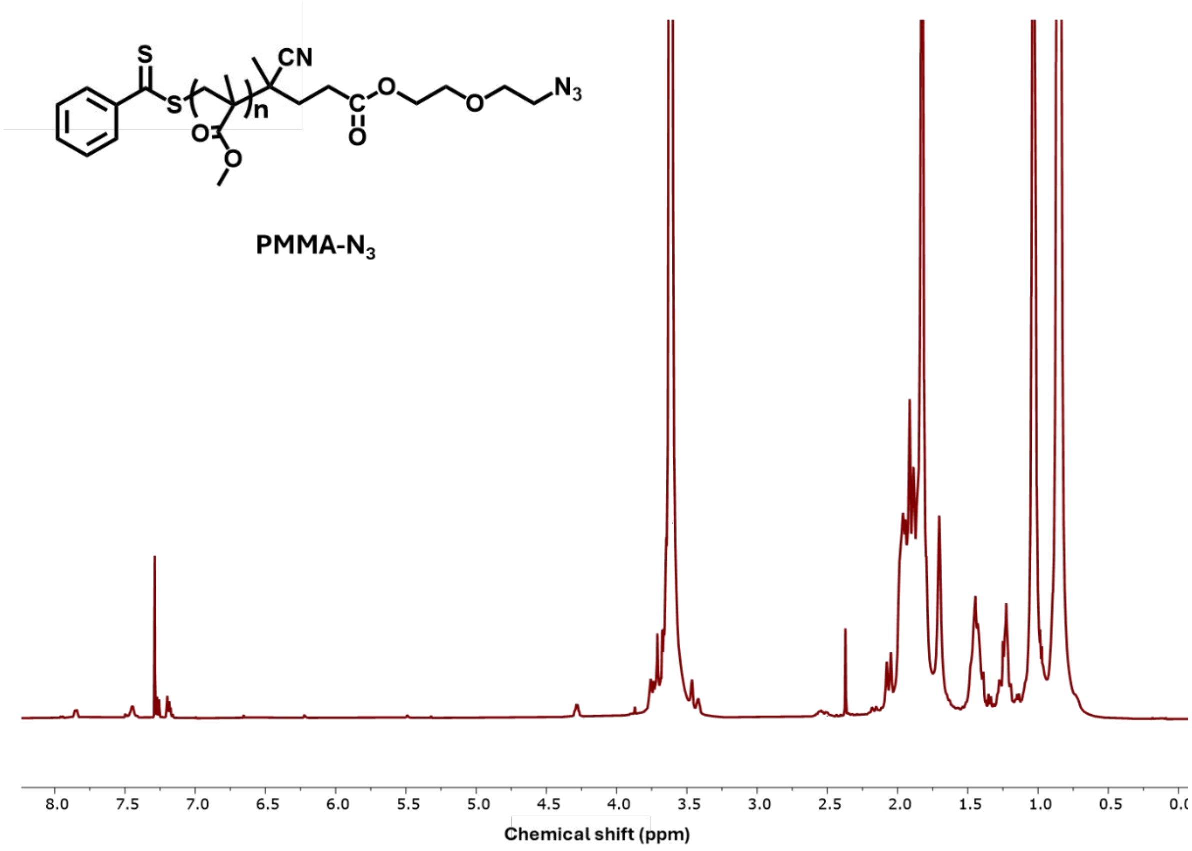

Figure S1r. ^{1}H NMR spectra of PMMA-N_3 in $CDCl_3$.

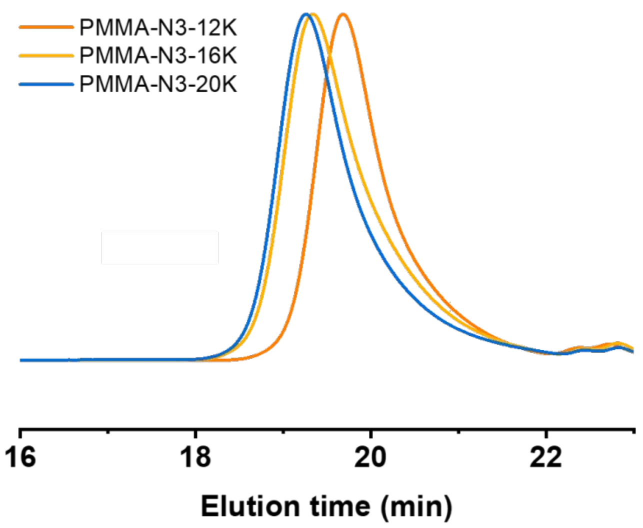

Figure S1s. SEC elution curve of PMMA-N_3 using THF as eluent.

Table S3. Molecular information of PMMA-N_3.

Polymers	M_n (kDa)[a]	***Đ***[b]
PMMA-N_3-12k	11.8 k	1.26
PMMA-N_3-16k	16.8 k	1.36
PMMA-N_3-20k	20.4 k	1.30
a Determined by ^{1}H NMR. *b* Determined by SEC-MALLS		

General procedure for click reaction of PMMA-DAT/Thy

Example of graft to synthesis of **PMMA-DAT**: A round-bottom flask equipped with a magnetic stir bar was charged with PMMA-N_3 (1.00 g, 0.085 mmol), DAT-alkyne (25.93 mg, 0.10 mmol)), PMDETA (35.25 mg, 0.20 mmol) and anhydrous THF (10 ml). The solution was degassed by bubbling dry nitrogen gas for 15 min, after which CuBr (29.18 mg, 0.20 mmol)) was added into the flask under a nitrogen atmosphere. The mixture was stirred at 40 °C for 24 hours, then cooled to room temperature. The crude product was purified by column chromatography on aluminum oxide (neutral) to remove the copper catalyst.

Most of the THF was subsequently removed under reduced pressure, and the remaining material was precipitated twice in hexane to afford PMMA-DAT as a pink solid. Successful synthesis was confirmed by ^{1}H NMR and SEC-MALLS analyses. In the ^{1}H NMR spectra, the disappearance of the peak at 3.4 ppm for PMMA-N_3 and the appearance of a new peak at 3.95 ppm for PMMA-DAT indicated complete conjugation of the DAT functional group. The SEC elution profile remained almost unchanged after the click reaction, confirming that no polymer chai degradation occurred.

The synthesis of PMMA-Thy followed the same procedure as that of PMMA-DAT, except that Thy-alkyne was clicked onto PMMA-N_3.

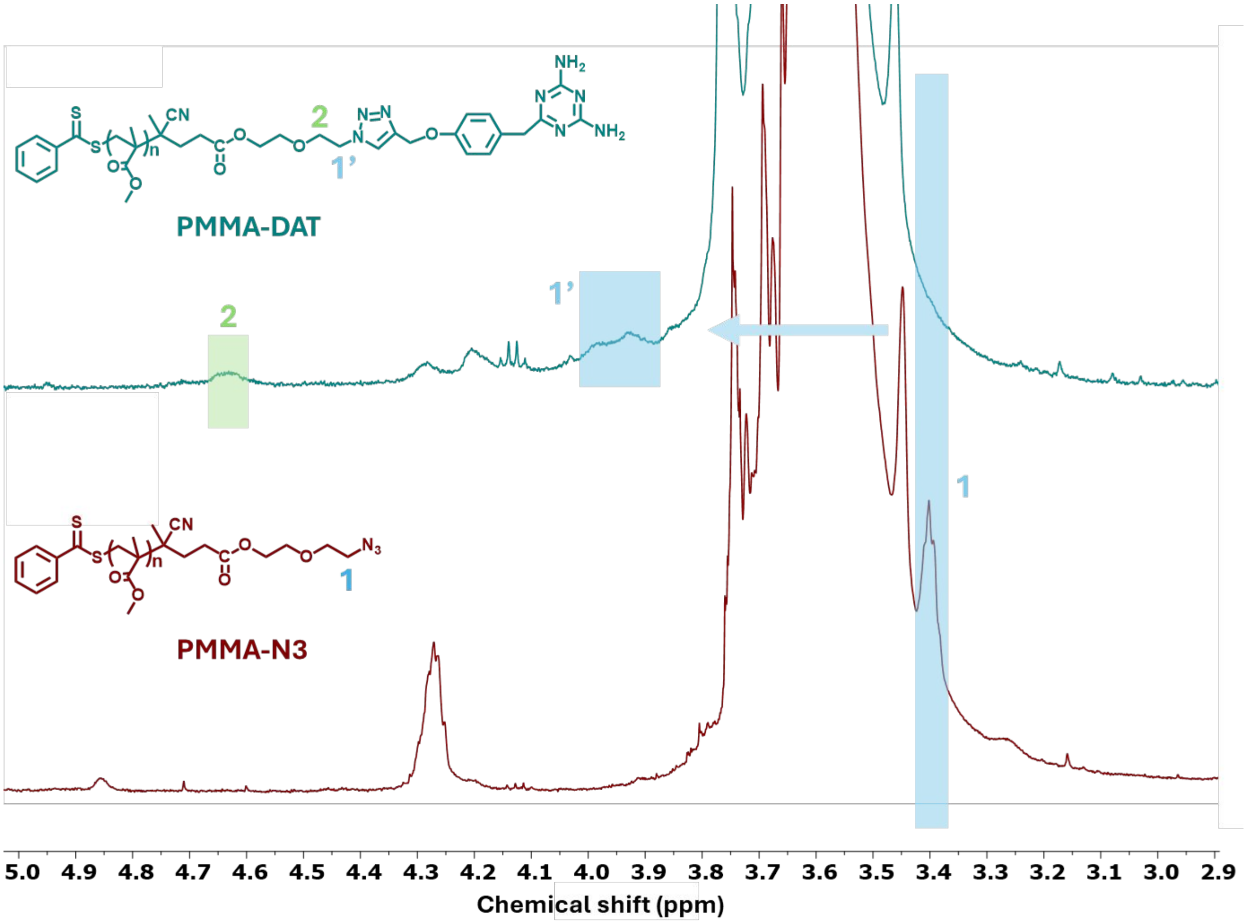

Figure S1t. ^{1}H NMR spectra of PMMA-N_3 and PMMA-DAT. The disappearance of the peak at 3.4 ppm (PMMA-N_3) and the appearance of a new peak at 3.95 ppm (PMMA-DAT) confirm the successful conjugation of the DAT functional group via the click reaction.

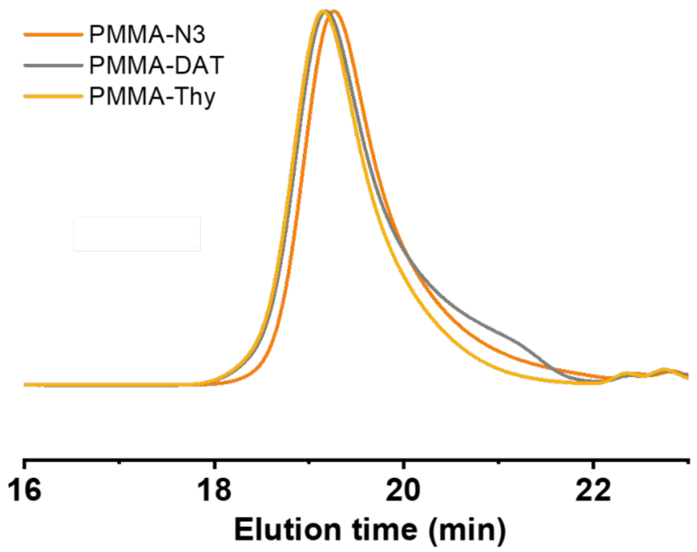

Figure S1u. SEC elution curves of PMMA-N_3 and PMMA-DAT using THF as eluent. The nearly identical elution profiles indicate no degradation of the polymer chain after the click reaction, confirming the structural integrity of the synthesized polymer.

<u>**Synthesis of PEG-DAT using 'grafting-to' method**</u>

PEG-DAT was synthesized by the azidation of commercially available mPEG-Br, followed by a click reaction with DAT-alkyne.

Take mPEG-5kDa as an example: A round-bottom flask equipped with a magnetic stir bar was charged with mPEG-5k (2.00 g, 0.4 mmol), sodium azide (1.04 g, 16 mmol) and H_2O (10 ml). The mixture was stirred at 45 °C for 48 hours. Upon completion, the reaction mixture was cooled to room temperature

then extracted with DCM (2 x 25 ml). The combined organic layers were dried over Na_2SO_4, and the solvents were removed under reduced pressure. The resulting product was precipitated in hexane to afford PEG-N_3 as a white solid.

The synthesis of PEG-DAT via the click reaction followed the same procedure as that for PMMA-DAT, except that DAT-alkyne was reacted with mPEG-N_3. PEG-DAT samples with molecular weights of 2.0, 3.4, 5.0, and 10.0 kDa were prepared. Successful synthesis was confirmed by ^1H NMR and SEC-MALLS. In the ^1H NMR spectra, the disappearance of the peak at 3.50 and 3.82 ppm for mPEG-Br and the appearance of a new peak at 3.70 and 3.41 ppm for PEG-N_3 indicated the complete azidation of PEG. The subsequent disappearance of the peak at 3.70 and 3.41 ppm for PEG-N_3 after the click reaction confirmed complete conjugation of the DAT functional group. The SEC elution curve profile remained nearly unchanged after the click reaction, indicating no degradation of the polymer chain.

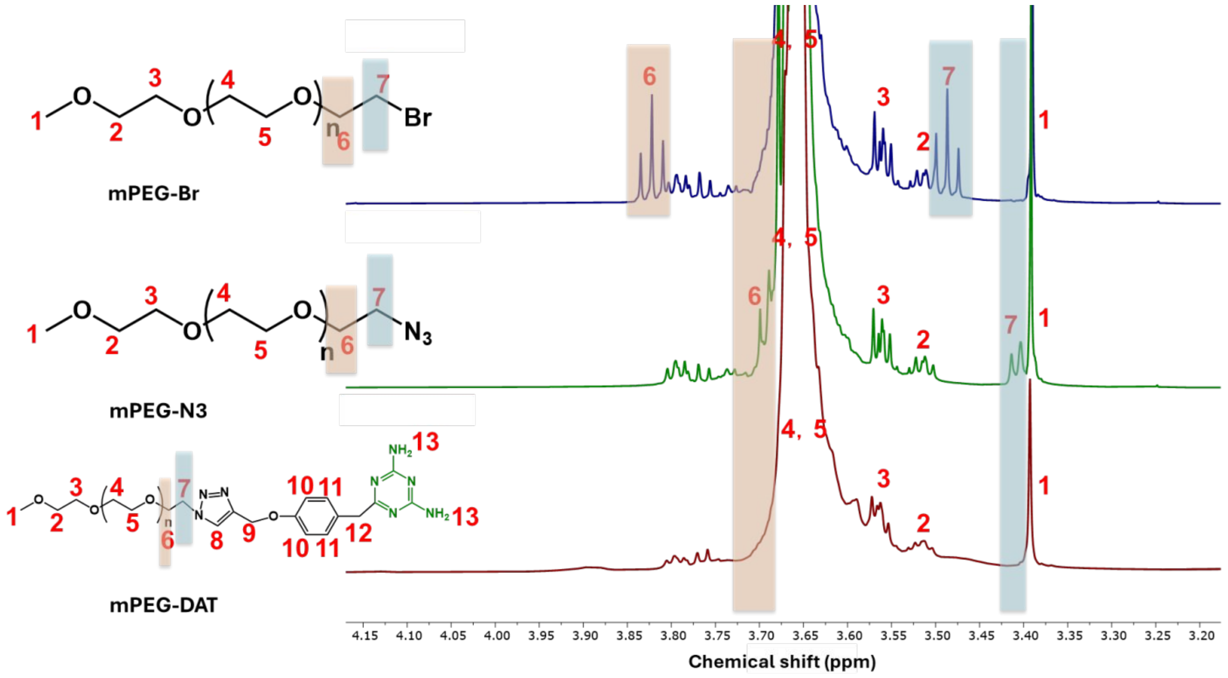

Figure S1v. ^1H NMR spectra of mPEG-Br, PEG-N_3 and PEG-DAT.

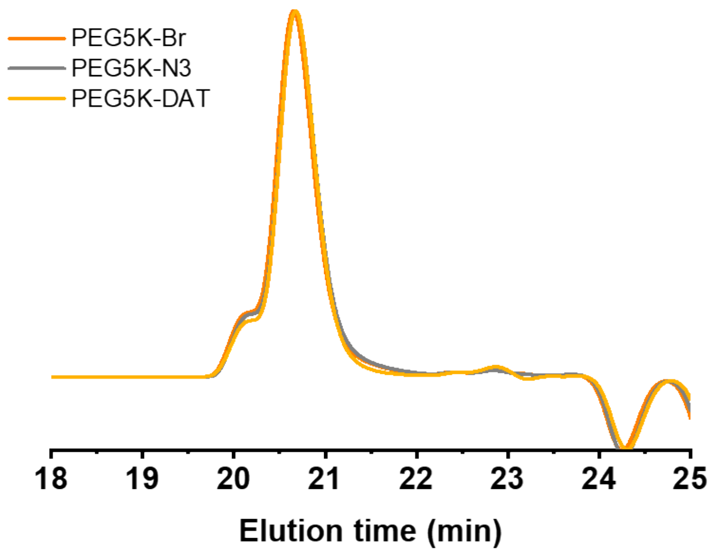

Figure S1w. SEC elution curves of mPEG-Br, PEG-N_3 and PEG-DAT using THF as eluent.

3. Automated and high-throughput fabrication of supramolecular block copolymers

All homopolymer precursors were dissolved in anhydrous toluene at an identical molar concentration of 1.54 mM, ensuring equimolar ratios between the DAT and Thy functional groups. Maintaining this uniform concentration was critical for reliably promoting effective intermolecular hydrogen bonding, thereby eliminating discrepancies that could arise from concentration variations and enabling precise, consistent self-assembly across the entire library. Using an OT-2 liquid-handling robot, equal volumes of each DAT-functionalized homopolymer solution and each Thy-functionalized homopolymer solution were combined to generate the full library of SPBs.

SPB thin films were subsequently spin-cast directly onto Si wafers at 2000 rpm for 1 min without preheating the solutions. Owing to the different mass concentrations, the resulting film thicknesses varied among the SPBs.

The SPB thin films were subsequently immersed in ethanol for 15 min, followed by drying with a nitrogen (N_2) stream, to perform a reconstruction treatment. Ethanol selectively dissolves the POEGMA phase, while polystyrene (PS) remains insoluble under these conditions. This selective removal enhances the contrast between the two domains and allows clearer visualization of the phase-separated morphology in AFM measurements. The reconstruction step is therefore used only to improve imaging contrast and does not alter the underlying domain structure.

4. Automated atomic force microscopy (AFM)

Automated AFM measurements were conducted using the Micro-builder module within the Igor-based Jupyter Asylum AFM software. During the initial AFM characterization, several scan sizes, including 5 × 5 μm, 1.5 × 1.5 μm, and 1 × 1 μm (the sample used in this preliminary study is not included in the present manuscript) were examined (**Figure S4**). A scan size of 1.5 × 1.5 μm provides a suitable balance between resolving the domain morphology and capturing a representative measurement area. For convenience in data analysis, a nearby value of 2 × 2 μm was used in the manuscript.

A custom-machined sample holder capable of accommodating 52 samples with fixed spacing was used to enable precise and reproducible stage translation between samples (**Figure S3**). Each sample was positioned at the center of its designated location in the holder. Because the spacing between all positions was identical, the AFM stage could move the same incremental distance between measurements, ensuring that imaging was consistently performed in the central region of each sample, thereby minimizing potential edge effects associated with spin-coated film non-uniformity.

At each sample position, the AFM tip first performed automatic alignment, followed by acquisition of an optical microscope image to verify the scanned region. AFM imaging was then performed automatically, and all images were systematically archived for downstream analysis.

To ensure reliable data quality, nine AFM images were collected for each sample at different positions. In practice, occasional imaging failures occurred due to surface defects or dust particles on the sample surface. On average, approximately one failed image occurred every 3–4 samples. All images were manually inspected, and those affected by artifacts (e.g., dust particles, scanning instability, or tip contamination) were excluded. The highest-quality image from each sample was then selected for further analysis.

The AFM probe tip was inspected after completing each batch of samples in the holder. If degradation in image quality was observed, the probe tip was replaced before starting the next batch. In practice, the tip was typically replaced once per batch.

Human intervention during the automated workflow was minimal. Samples were manually loaded between batches, after which the imaging process proceeded automatically. If automated scanning repeatedly failed for a given sample, that sample was flagged and subsequently measured manually.

The complete AFM imaging of all samples required approximately one week. Loading and transferring a new batch of samples typically required ~1 hour of manual work, after which the system ran automatically. If a batch finished during nighttime hours, the next batch was loaded the following morning, which contributed to the total elapsed time. No additional system maintenance was required during the measurement period.

5. Morphology explanation of other SPBs

In addition to the POEGMA/PS SPBs system, we also looked into other material systems. Other SPBs systems did not exhibit clear nano-structural features, likely due to multiple factors. For SBCPs containing PEG segments, PEG crystallization inhibited the fabrication of SBCPs, as evidenced by crystallite peaks in 1D GIWAXS profiles and large aggregates observed in AFM (**Figure S7, 8 and 10**). In

addition, the relatively low χ values between PMMA and PS, as well as between PMMA and POEGMA, rendered low-MW polymers in these systems largely miscible, preventing distinct microphase separation.

6. AFM image binarization and domain spacing extraction

To process AFM images in a high-throughput manner, a Python script was developed in-house for image binarization and domain spacing quantification. Because of the time and labor cost associated with manual labelling of images, AFM images were down sampled to a data subset consisting of one image per combination of molecular weight values considered representative of images for that sample. The curated data subset consists of 117 images. The images were then binarized using adaptive binarization, Niblack binarization, manual segmentation, or some combination of two or more methods, as deconstructed in **Figure 3e**, with the goal of separating minority and majority phases.

Adaptive and Niblack binarizations were performed using the OpenCV library, but parameter tuning for thresholding and preprocessing was implemented with a custom interactive widget, implemented in Jupyter Notebook and made available on our Github page. Manual annotations were performed using the open-source software Labelme using a combination of hand-drawn polygons and AI segmentation with the built-in EfficientSam tool.[5] "Combination" binarizations are accomplished by one of two scenarios: either by preliminary segmentation with the binarization widget later fine-tuned manually, or through a logic combination (AND/OR) of adaptive and Niblack binarizations, which can be implemented through the binarization widget. Combining masks in this way is especially advantageous for microscopy images with features at multiple scales within the same image where applying only a single filter with a fixed block size can fail to capture the full range of feature sizes.

With the binarized images, a Voronoi analysis was performed, using each minority phase element as the seed of a Voronoi region. The Voronoi ridge is composed of the pixels in the majority phase equidistant to two or more distinct minority phase domains as determined by Euclidean distance transform

over the binarized image. For every pixel along the ridge, twice the Euclidean distance to the nearest minority phase feature is binned into a histogram, and the mean of that distribution taken as the domain spacing for the image.

7. Machine learning-guided morphology prediction and inverse SPB design

Independent, single-output regression ML models were trained to predict the log distribution mean for Voronoi-measured images. To simplify the downstream inverse design task, only molecular weights $M_{N,\,POEGMA}$ and $M_{N,\,PS}$ were used as features. Results for trained model types including linear, power, random forest regressor (RFR), multilayer perceptron (MLP), extreme gradient boosting regressor (XGB), support vector regressor (SVR), and Gaussian Process Regressor (GPR) are shown in **Table S4** and **Figure S14**. Tabulated results are the best model (based on coefficient of determination, R^2) from hyperparameter random search with 10 repetitions of 5-fold cross-validation (CV), where repeated k-fold CV was used to suppress effects of the dataset's sensitivity to train-validation splits. And because the model lacks any chemical context and is therefore not intended nor expected to generalize. Note that unitless scores (R^2) are reported from fitting in log space, while scores with units (RMSE, MAE) are reported from real-space fits after exponentiating.

ML models remain imperfect at predicting target phase separation lengths and are susceptible to dataset splitting, highlighting potential imperfections in the dataset itself. Issues with the training data may arise from several sources: noisy data or non-representative images for a given sample due to the highly local nature of AFM, dataset imbalance, inaccurate or incorrect binarizations associated with ambiguity about imaged features and the difficulty of segmenting images with complex features, or shortcomings of the phase separation distance metric used. Additionally, small dataset size is a likely contributor to highly variable results from data splitting as well as model tendencies to underfit at higher phase separation lengths as seen in **Figure 5a** and **Figure S17**.

Given the established influence of volume fraction on phase separation behavior in block copolymer systems, the predicted phase separation lengths from SVR are also plotted as a function of $M_{N, PS}$ in **Figure 4c**. It can be observed that multiple constitutional combinations (molecular weights) can be used to obtain the same phase separation spacing, suggesting a high-degree of tunability in SPB systems based on the combination of inputs is possible based on the desired outcome material properties.

Trained models create an $\mathbb{R}^3$ manifold, which has been simply visualized for the SVR model as a heatmap in **Figure 4b** by performing a grid search over 50 values of each $M_{N, PS} \in [5000, 25000]$ kDa sampled at intervals of 408.2 kDa and $M_{N, POEGMA} \in [4000, 32000]$ kDa sampled at intervals of 571.4 kDa. Both SVR and RFR model manifolds are shown in **Figure 4a** and **Figure 5b**, respectively.

ML models enable informed design of materials with predictable properties from modelling without the need to synthesize the exact material *a priori*. To demonstrate predictive design in the context of SPBs, and to further validate the model in a real-world setting, several target phase separation values were chosen: 50, 100, and 150 nm. The SVR and RFR heatmaps were searched for values at each of these targets with a tolerance of ± 50 nm, and those highlighted combinations are shown in **Figure 4d and Figure S17**.

Due to material availability at the specific molecular weights required, the nearest available combination of molecular weights and not used for the training set were found for each target and three in **Table S5, 6** Actual RFR model predictions—those made from the model directly for the available molecular weight combinations rather than the heatmap—were 51, 96, and 152 nm for the 50, 100, and 150 nm targets, respectively. Predictions from the SVR model were 59, 85, and 174 nm. Both RFR and SVR predictions are tabulated in **Tables S5** and **S6**. AFM images of the blends were characterized by the same methods as previously described, with measured phase separation lengths using the mean of Voronoi ridge distance. The synthesized and measured images are shown in **Figure 4**, **c-e** for the RFR and in **Figure S17, c-e** for the SVR.

There is still room for model improvement. Much of the phase separation information is lost by compressing the spatial distribution to a single statistic for model training. Further efforts to improve image segmentation accuracy are also critically important for creating effective ML models with microscopy data. This model uses a small and imbalanced dataset of less than 120 datapoints and performance can be expected to improve with further integration of high throughput workflows and data curation with ML. Expanding the regression model to consider additional factors, including but not limited to broader molecular weight ranges, additional chemistries, sample processing parameters, polydispersity, and synthetic accessibility can unlock even higher-performing and more application-specific models. Finally, more sophisticated routes to inverse design like Bayesian optimization with active learning suggest an obvious route toward closed-loop accelerated workflows building upon these foundations.

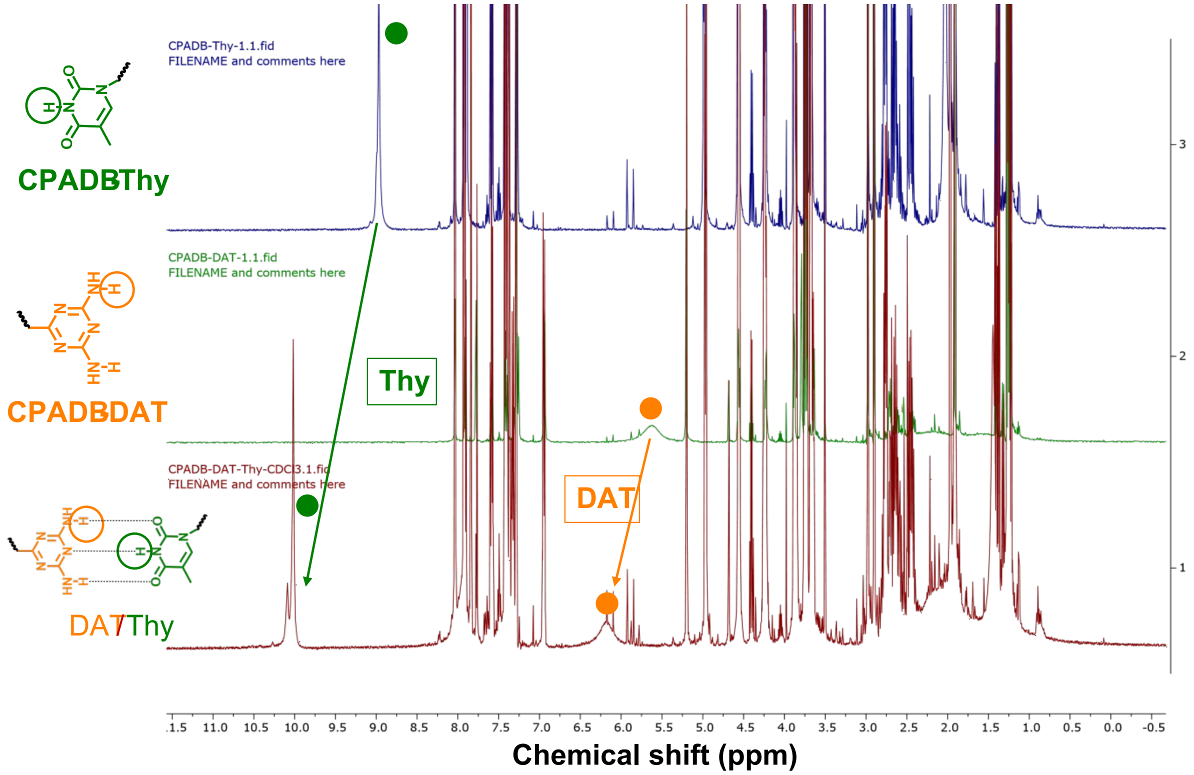

Figure S2. ^{1}H NMR spectra of CPADB-DAT, CPADB-Thy, and the blended CPADB-DAT/CPADB-Thy in $CDCl_3$. The observed upfield shifts of the DAT and Thy protons after blending indicate the successful formation of intermolecular hydrogen-bonding interactions between DAT and Thy.

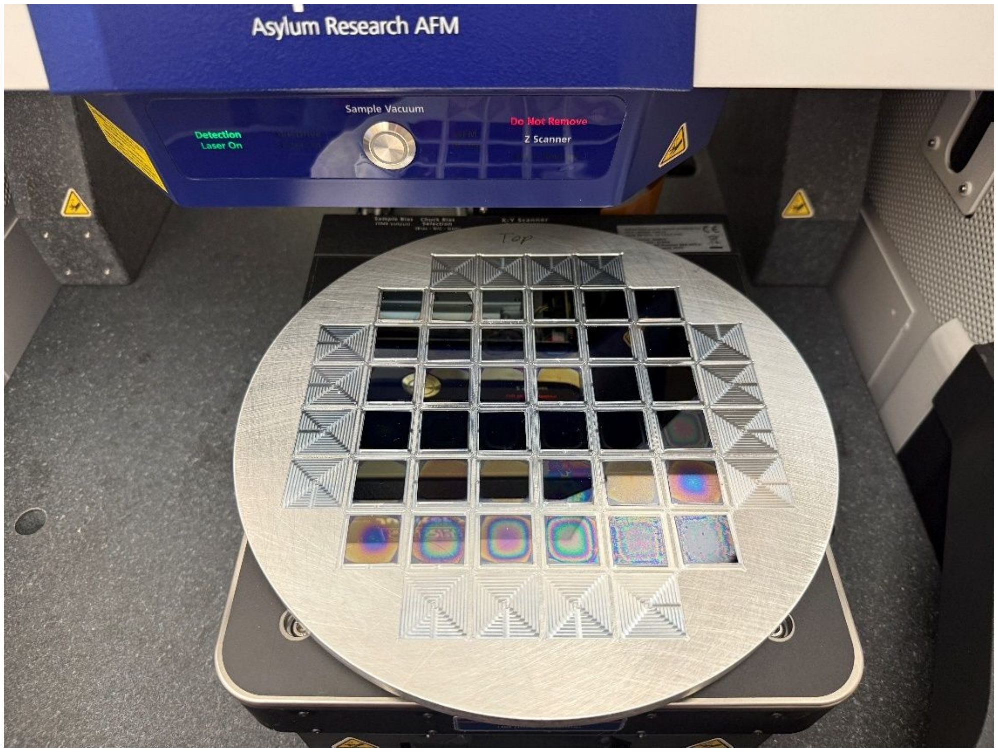

Figure S3. Photograph of customized sample holder for facilitating high throughput AFM measurements.

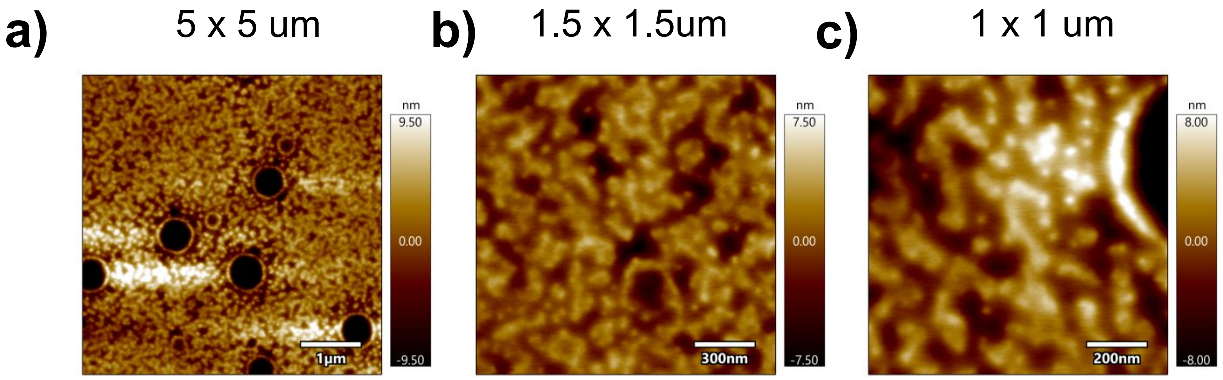

Figure S4. AFM images of the sample acquired at different scan sizes to evaluate the effect of imaging area on the observed morphology. Images were collected at scan sizes of (a) 5 × 5 µm, (b) 1.5 × 1.5 µm, and (c) 1 × 1 µm.

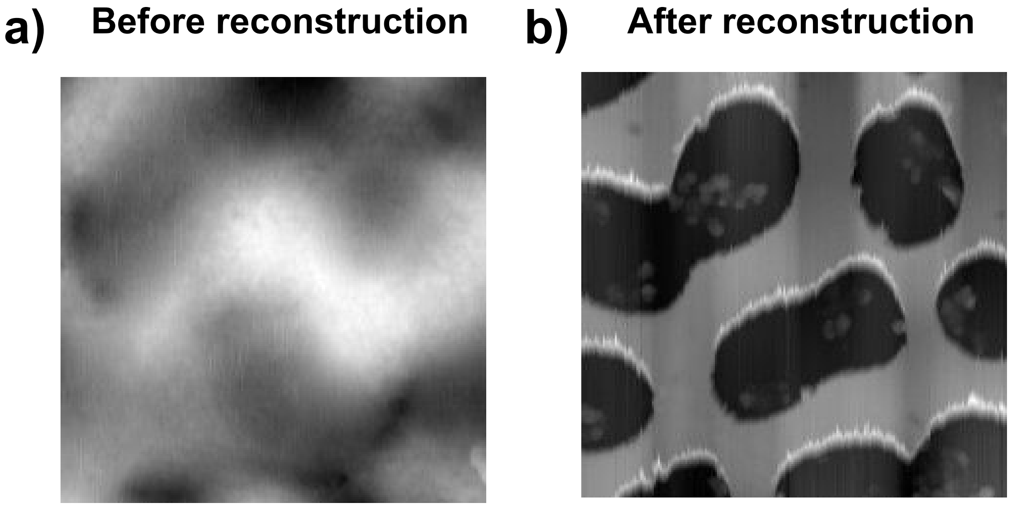

Figure S5. AFM images of the POEGMA/PS supramolecular polymer blend (SPB, MW 31 kDa–13.5 kDa) before and after the reconstruction treatment, showing that the overall domain size and morphology remain consistent. Thage image is 2um by 2um.

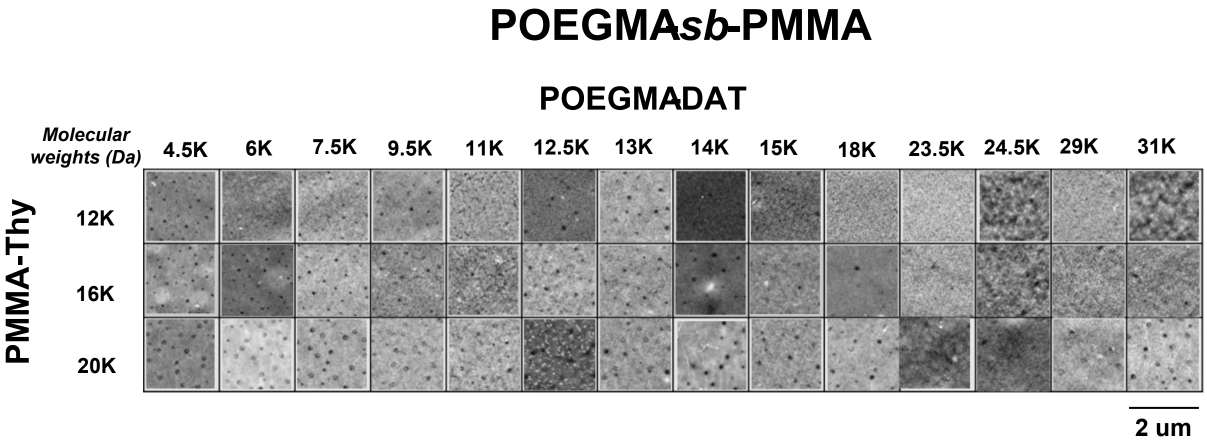

Figure S6. AFM height images of POEGMA-*sb*-PMMA after reconstruction in ethanol. The scan size is 2x2 um.

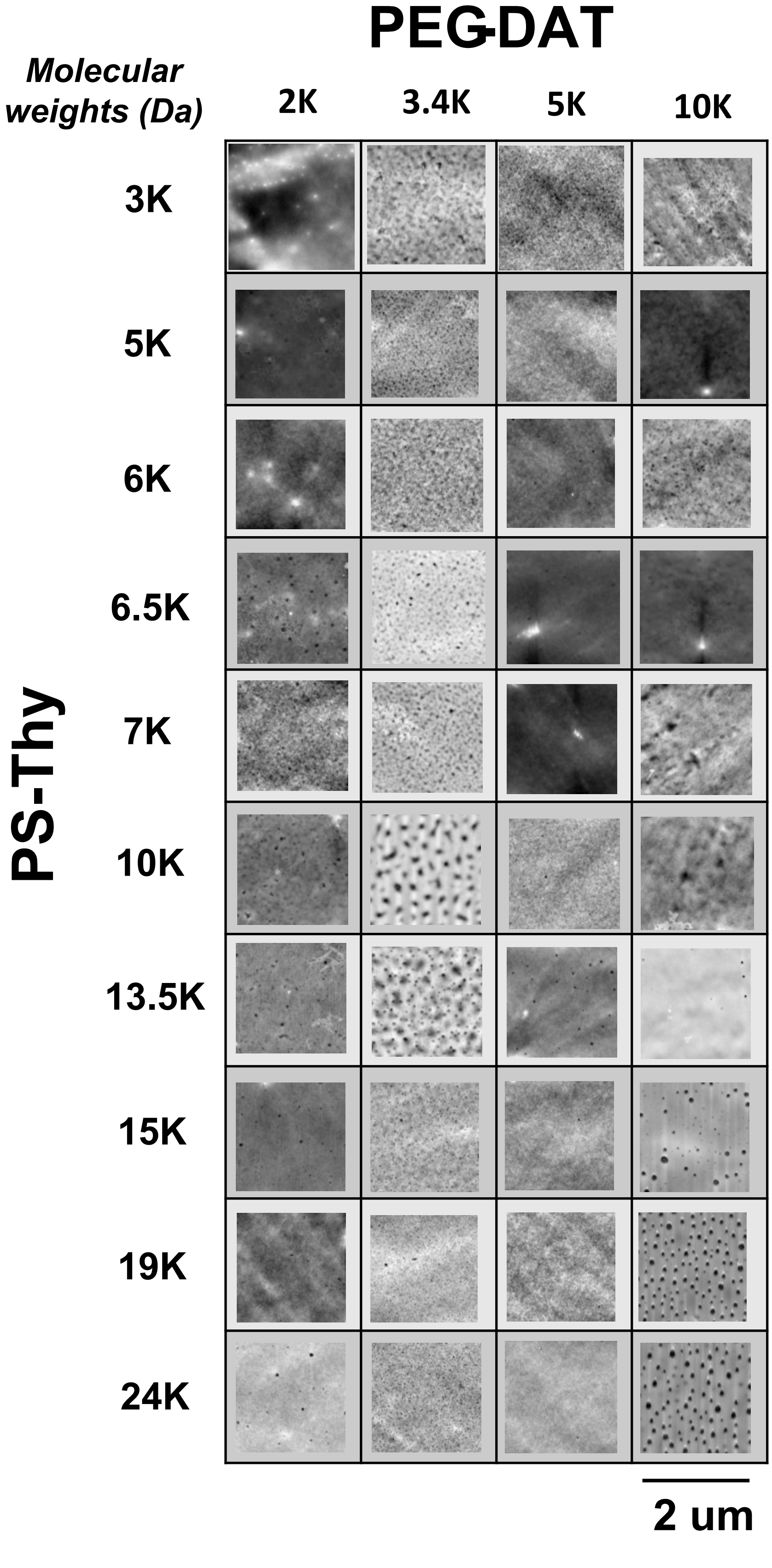
PEG-DAT
Molecular weights (Da)
2K
3.4K
5K
10K
PS-Thy
3K
5K
6K
6.5K
7K
10K
13.5K
15K
19K
24K
2 um

Figure S7. AFM height images of PEG-*sb*-PS. The scan size is 2x2 um.

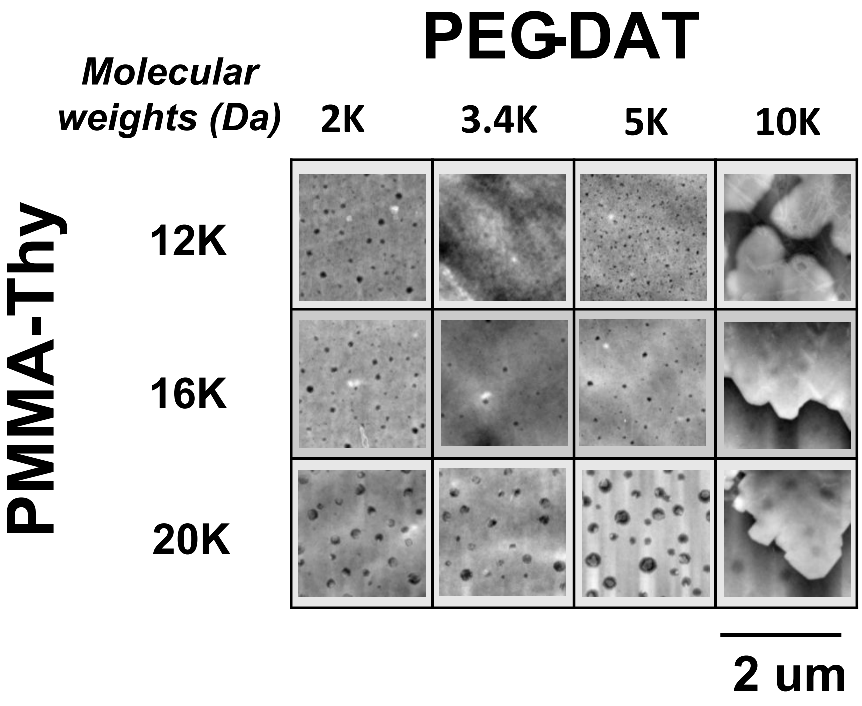

Figure S8. AFM height images of PEG-*sb*-PMMA. The scan size is 2x2 um.

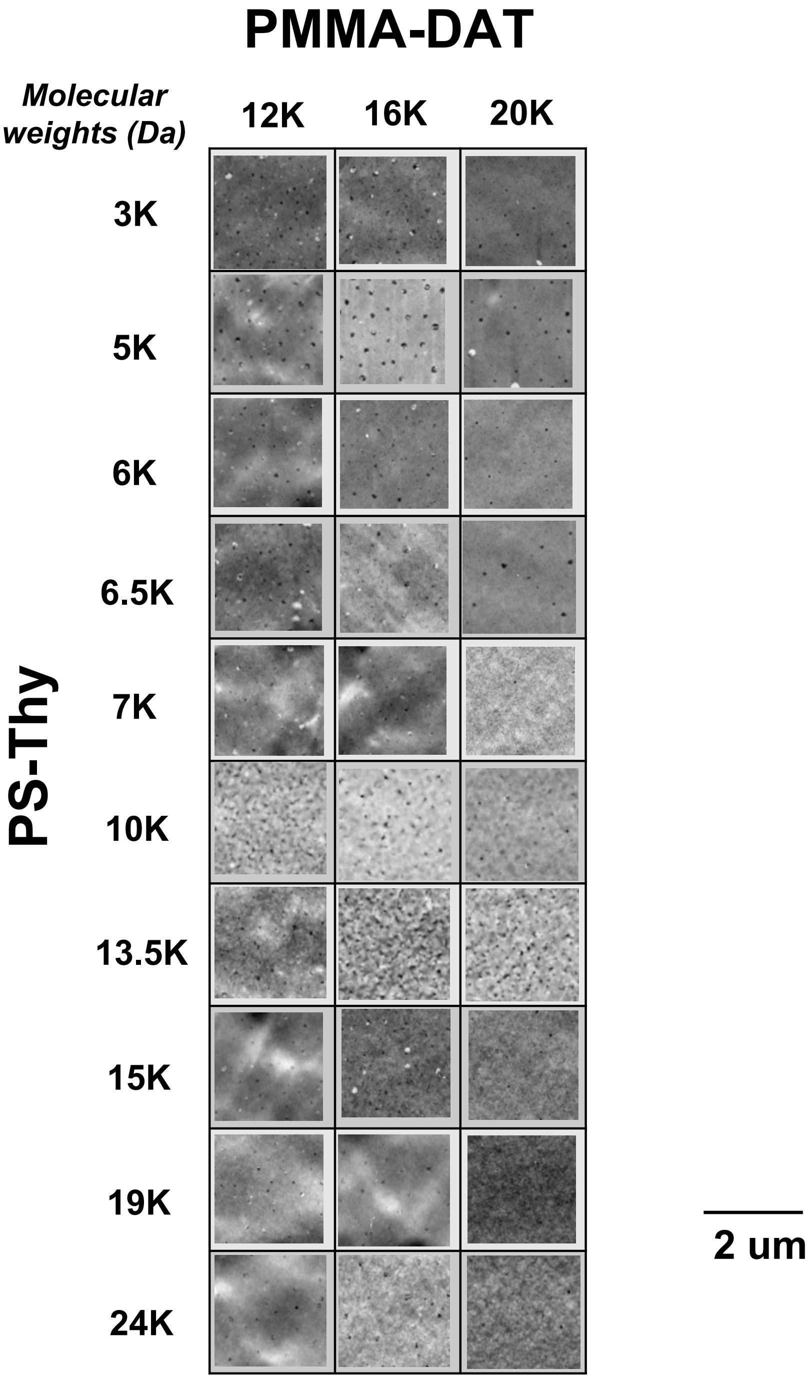

Figure S9. AFM height images of PMMA-*sb*-PS. The scan size is 2x2 um.

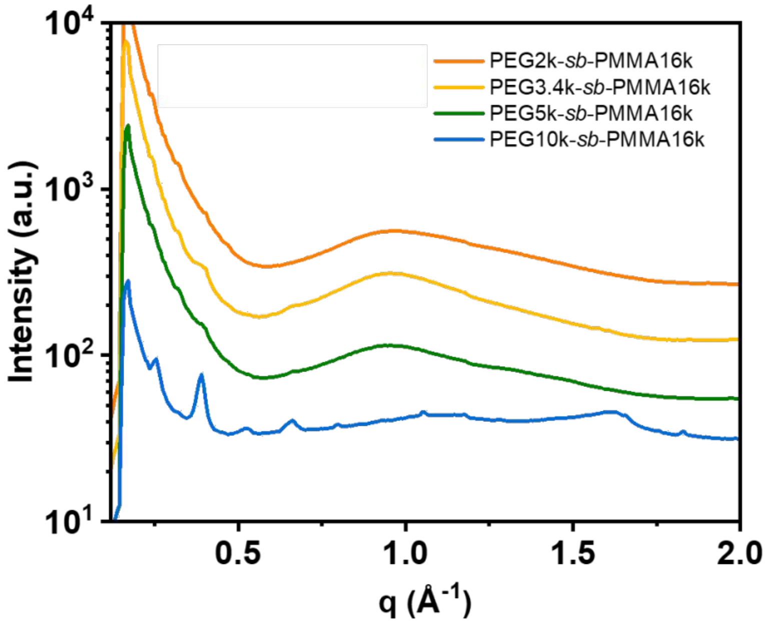

Figure S10. 1D scattering profiles of PEG-DAT-2k-*sb*-PMMA-Thy-16k, PEG-DAT-3.4k-*sb*-PMMA-Thy-16k, PEG-DAT-5k-*sb*-PMMA-Thy-16k and PEG-DAT-10k-*sb*-PMMA-Thy-16k from grazing-incidence wide-angle X-ray scattering.

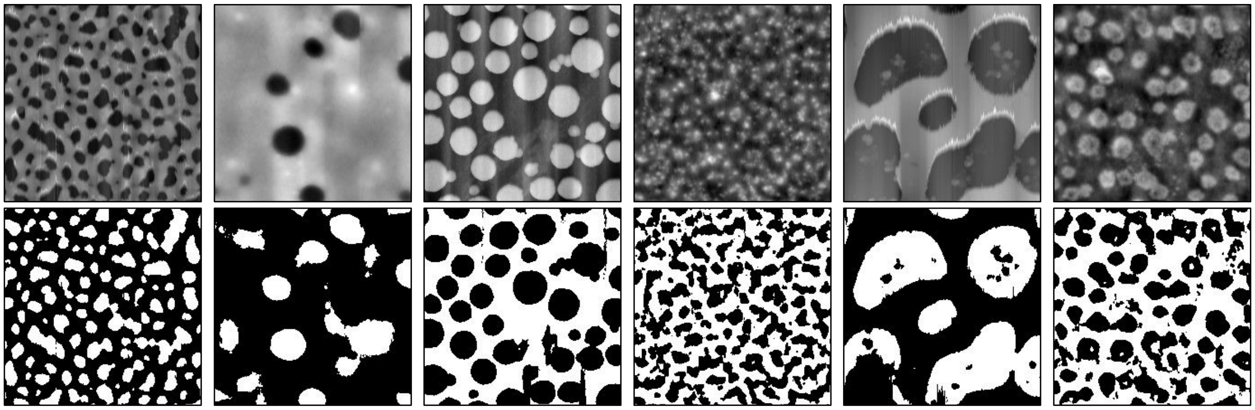

Figure S11. AFM images (top) and their binary mask counterparts (bottom) when using the same binarization parameters for each (mean adaptive threshold, block_size=95, C=14).

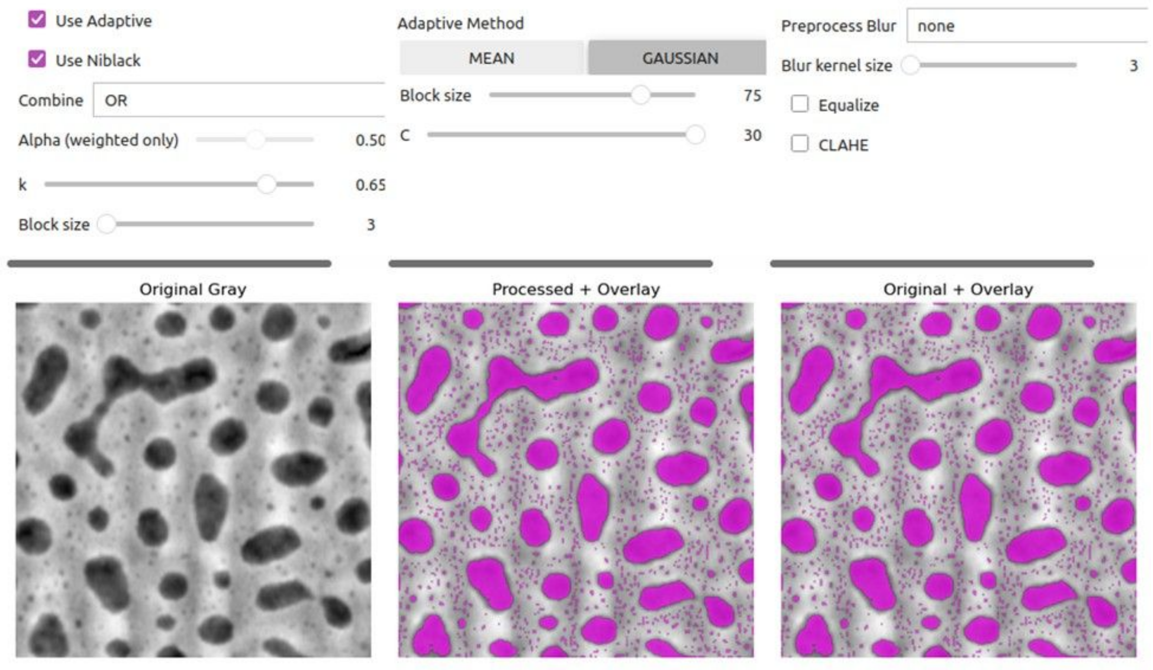

Figure S12. User interface for the custom binarization widget. Masks are generated in real-time in response to changing parameters. Logic combination of binarization algorithms can capture multiple length scales simultaneously for images with complex features. Generated mask shown for demonstration, not used for image analysis/model training.

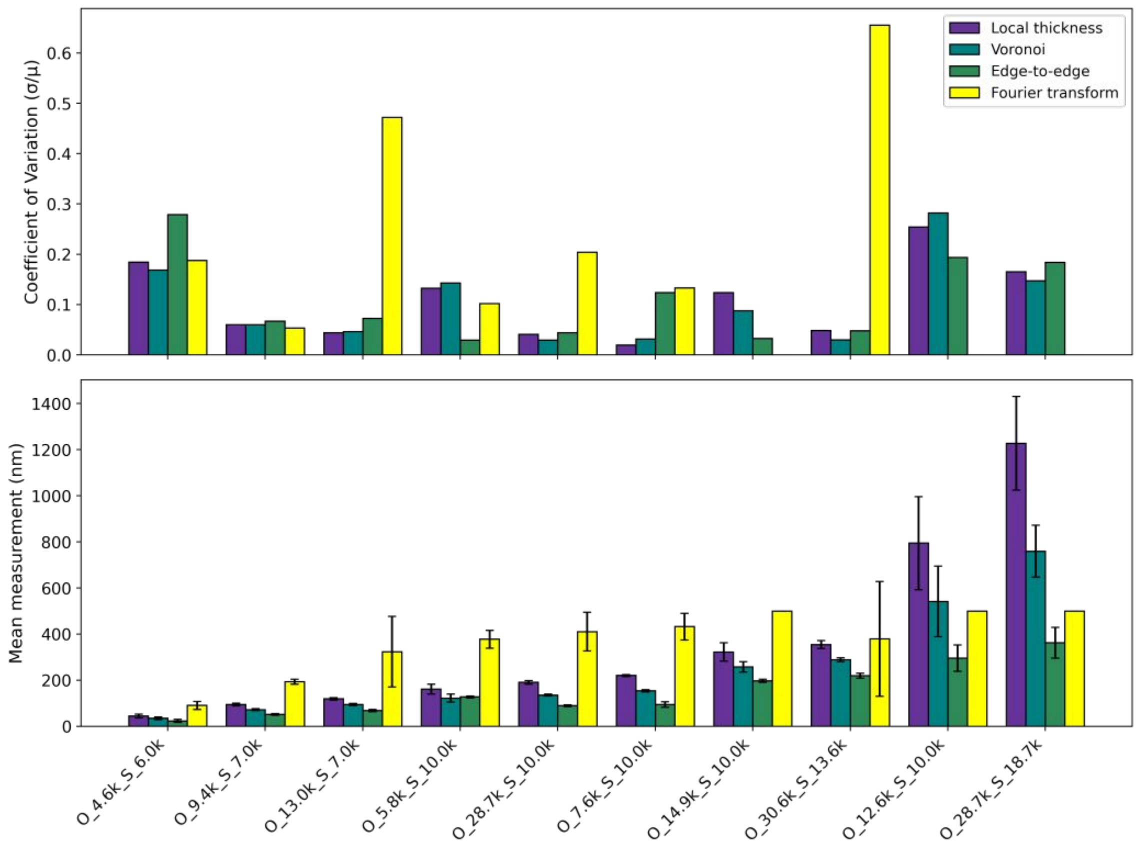

Figure S13. Comparison plots for phase separation length metrics on a subset of samples. Top: coefficient of variation for three images from each sample when using the same binary mask. Bottom: average measurement of mean phase separation length for each sample and analysis combination. Error bars are standard deviation of the measured images for each sample (n=3).

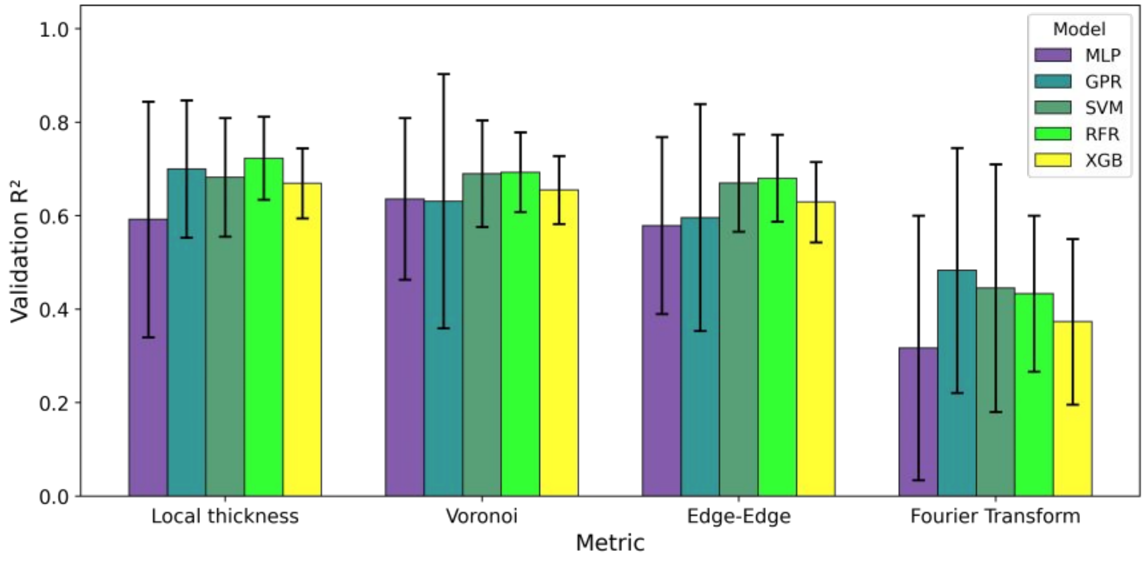

Figure S14. Model performances compared using several different model architectures for different approaches to quantifying phase separation length. Values are the average validation R^2 across 10 repeats of 5-fold cross-validation. Error bars are the standard deviation for those 50 validation sets.

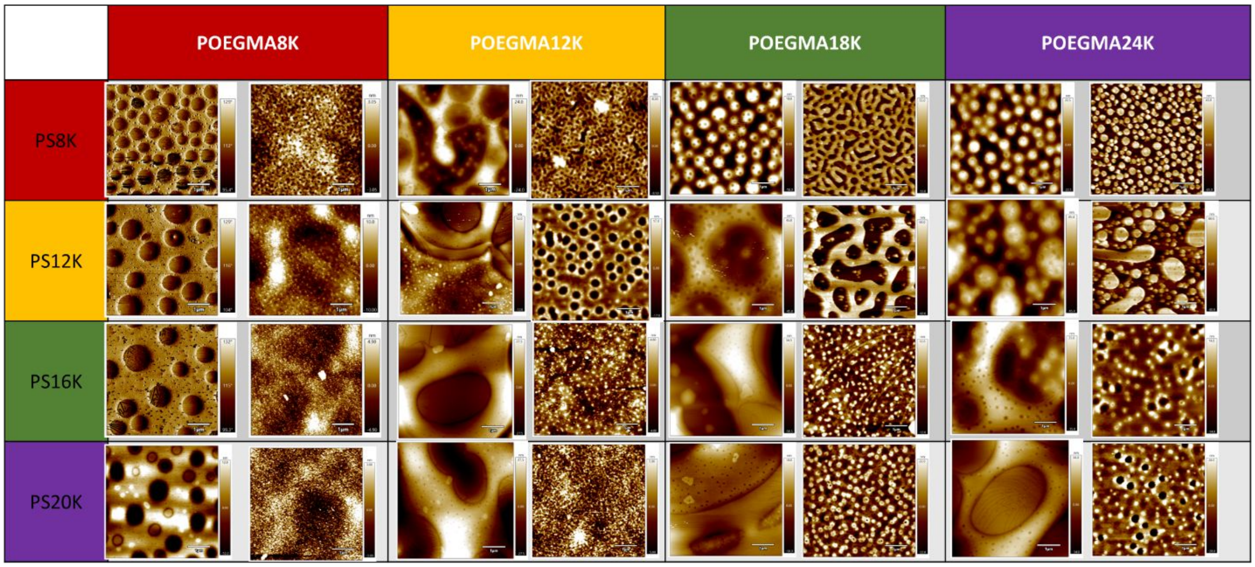

Figure S15. AFM height images of control blended samples with or without hydrogen bonding. POEGMA-DAT/PS (no hydrogen bonding as regular PS without hydrogen bonding end group were used) with POEGMA-sb-PS SBCPs with similar MWs. For each MW, the left one is the samples without

intermolecular hydrogen bonding interactions whereas the right one is the SPBs. The scan size is 5 um by 5 um.

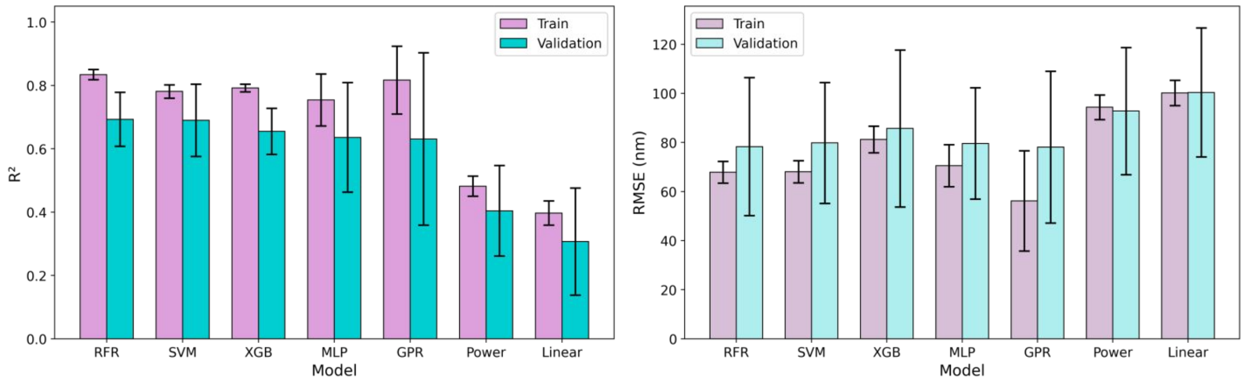

Figure S16. ML model performance. a) R^2 values for both train and validation set across 10 repeats of 5-fold cross-validation. Error bars are the standard deviation from all rounds (n=200 for train, n=50 for validation). b) RMSE values for both train and validation sets across 10 repeats of 5-fold cross-validation.

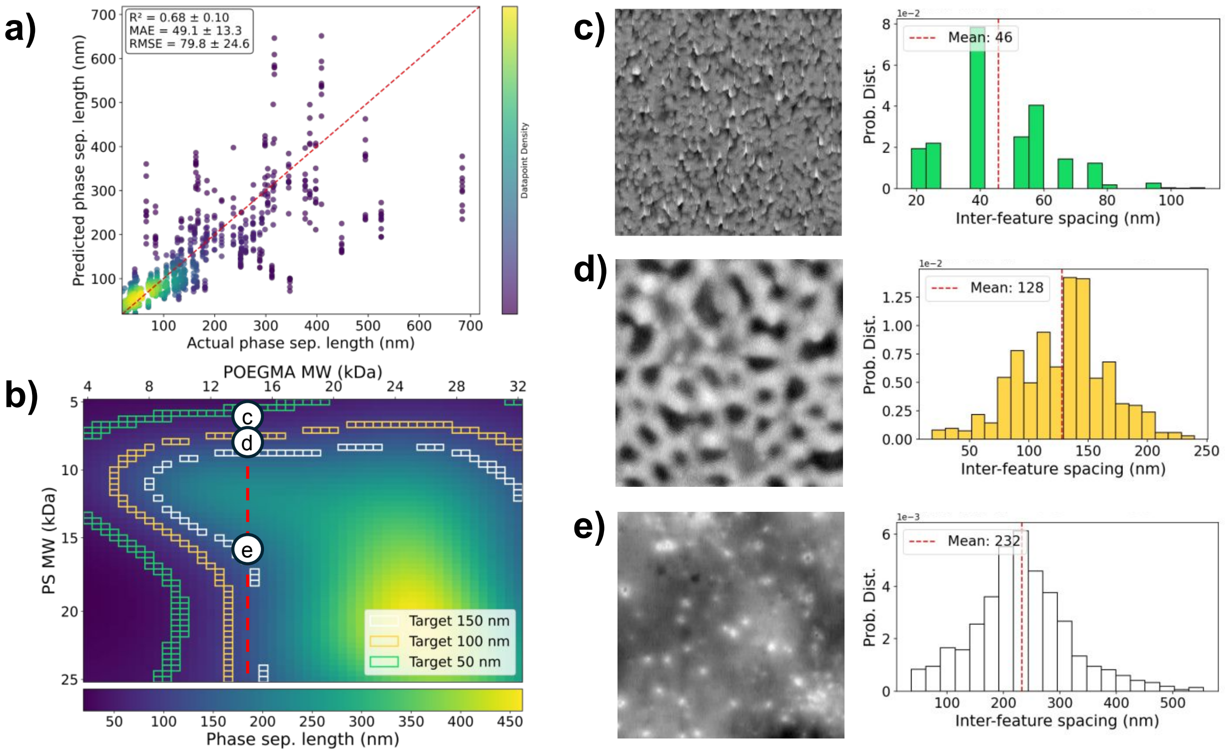

Figure S17. ML–based morphology prediction and inverse design of SPBs**.** (a) Parity plot of measured versus predicted domain spacings from all validation data points across 10 repeats of 5-fold CV training the SVR model. Z-axis colors denote relative density of datapoints. (b) SVR prediction heatmap highlighting compositions within ± 5 nm of selected targets (50, 100, and 150 nm). Experimental validation for inverse design at these targets is shown in (c) 50 nm, (d) 100 nm, and (e) 150 nm.

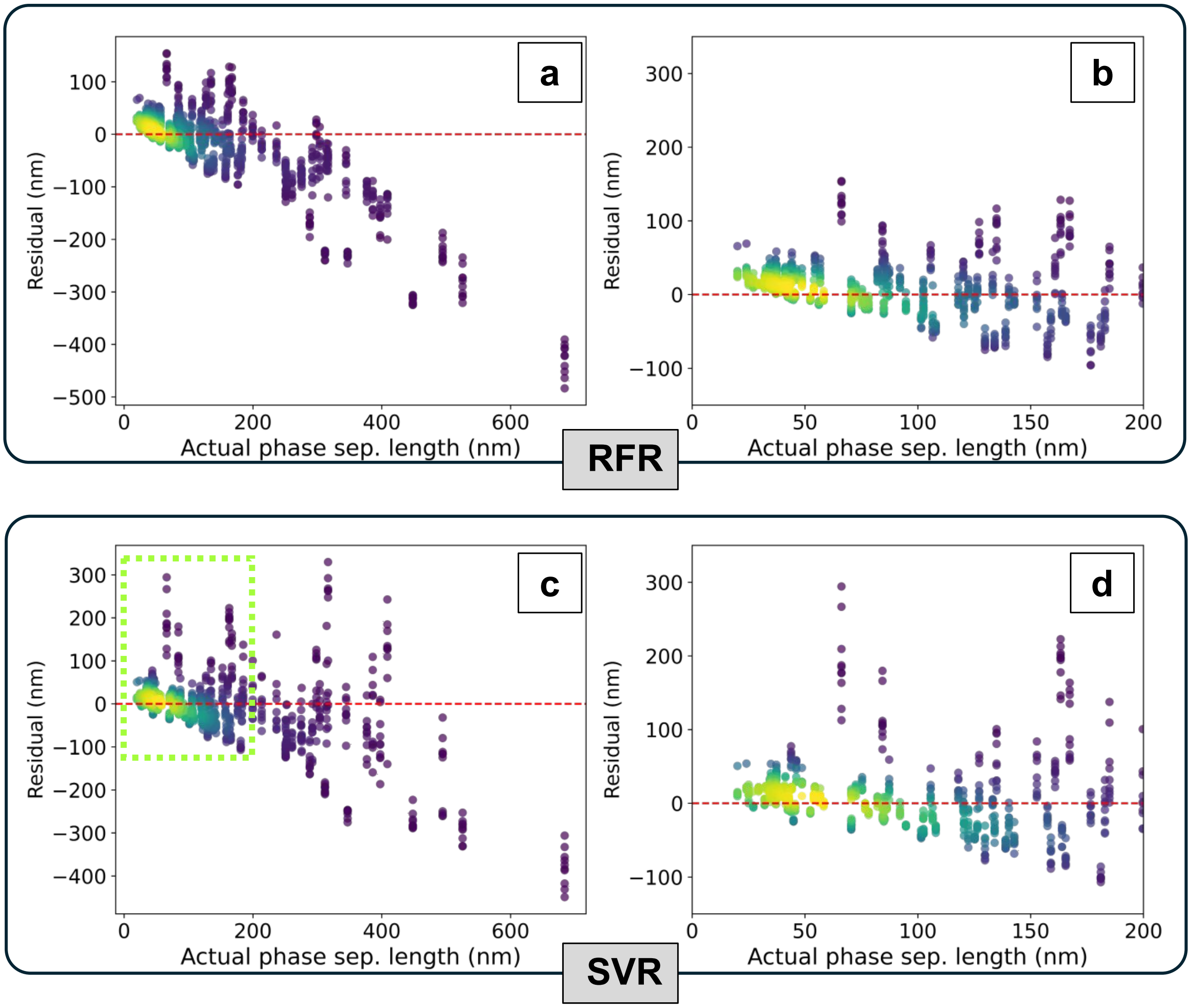

Figure S18 a, c) Residual plots for the RFR and SVR models. Z-axis color is coded according to datapoint density. b, d) the x-range [0, 200] for each residual plot (shown by the green box in a, c) in expanded view.

Table S4. R^2 and root mean squared error (RMSE) values for models trained when predicting each of the phase separation distance measurement methods. Error is standard deviation from 10 repeats of 5-fold CV.

**Performance**	**Linear**	**Power**	**RFR**	**SVR**	**XGB**	**MLP**	**GPR**
Validation R^2 (nm^2/nm^2)	0.307 ± 0.169	0.404 ± 0.143	0.693 ± 0.085	0.690 ± 0.114	0.655 ± 0.073	0.636 ± 0.173	0.631 ± 0.272
RMSE (nm)	100.4 ± 26.3	92.8 ± 25.9	78.3 ± 28.1	79.8 ± 24.6	85.7 ± 32.0	79.0 ± 22.7	78.1 ± 30.9

Table S5. M_N combinations were identified for several phase separation length targets, combinations were down-selected to those shown based on material availability. Actual samples made along with their SVR model predicted phase separation lengths are shown alongside measured values from computer vision.

**Target (nm)**	**50**	**100**	**150**		Error between	MAE (nm)
Suggested PS Mn (kDa)	5.8	7.4	16.0			
Actual PS Mn (kDa)	6.0	7.0	15.2		Target: actual	38
Measured phase sep (nm)	46	128	232		Prediction: actual	38
Predicted phase sep (nm)	59	85	174		Target: prediction	16

Table S6. Suggested combinations and performance for RFR model predictions.

**Target (nm)**	**50**	**100**	**150**		**Error between**	MAE (nm)
Suggested PS Mn (kDa)	5.0	6.2	18.9			

Actual PS Mn (kDa)	5.0	6.2	18.7		**Target: actual**	15
Measured phase sep (nm)	49	105	111		**Prediction: actual**	17.3
Predicted phase sep (nm)	51	96	152		**Target: prediction**	2.3

References

1. Herbst, F. & Binder, W. H. Comparing solution and melt-state association of hydrogen bonds in supramolecular polymers. *Polym Chem* **4**, 3602–3609 (2013).
2. Edward Lindsell, W., Murray, C., Preston, P. N. & Woodman, T. A. J. Synthesis of 1,3-diynes in the purine, pyrimidine, 1,3,5-triazine and acridine series. *Tetrahedron* **56**, 1233–1245 (2000).
3. Wang, Y. & Wei, H. Synthesis and Properties of Cyclic Thermo-responsive Double Hydrophilic Block Copolymers. *Acta Polymerica Sinica* **50**, 291–299 (2019).
4. Abdelhamid, D., Arslan, H., Zhang, Y. & Uhrich, K. E. Role of branching of hydrophilic domain on physicochemical properties of amphiphilic macromolecules. *Polymer Chemistry* **5**, 1457–1462 (2014).
5. Xiong, Y. *et al.* EfficientSAM: Leveraged Masked Image Pretraining for Efficient Segment Anything. *Proceedings of the IEEE Computer Society Conference on Computer Vision and Pattern Recognition* 16111–16121 (2023) doi:10.1109/CVPR52733.2024.01525.
